# Supplementary material for: Refined efficacy estimates of the Sanofi Pasteur dengue vaccine CYD-TDV using machine learning
Source: Nat Commun. 2018 Sep 7;9:3644. doi: 10.1038/s41467-018-06006-6 (PMC6128884; doi:10.1038/s41467-018-06006-6)
Supplement: Supplementary file 1 — Supplementary Information [file 41467_2018_6006_MOESM1_ESM.pdf]

Supplementary Information | Refined efficacy estimates of the Sanofi Pasteur dengue vaccine CYD-TDV using machine learning

Dorigatti I et al.

1 Supplementary Methods ………………………………………………………………………………………………. pages 2-7

2 Supplementary Figures and Tables ……………………………………………………………………………….. pages 8 - 53

3 Supplementary References ……………………………………………………………………………………………. page 53

1. Supplementary Methods

1.1 Data

The dataset is comprised of the phase-2b (CYD23)[1] and the phase-3 (CYD14 and CYD15)[2,3] CYD-TDV trials, totalling 35,020 subjects. The dataset comprises 135 cases and 3,867 non-cases in CYD23, 598 cases and 9,609 non-cases in CYD14 and 664 cases and 20,147 non-cases in CYD15. Among the 35,020 subjects, 11,659 (33%) were assigned to the control arm and 23,361 (67%) to the vaccine group, reflecting the 2:1 vaccine to placebo randomisation used in the trials.[1-3] The female to male ratio was 1.04, with 17,871 females and 17,149 males enrolled in the trials and the average age at baseline was 10.91 [95% CI: 10.88, 10.95] years. Among the cases, 463 subjects were infected by DENV1, 407 by DENV2, 247 by DENV3, 230 by DENV4 and 50 subjects had missing information on the infecting serotype. On average, cases in the vaccine group were infected 48.6 [95% CI: 37.5, 59.7] days post-dose 3 (PD3). The PD3 titres (log-scale) were on average 2.13 [95% CI: 2.11, 2.16] against DENV1, 2.34 [95% CI: 2.31, 2.36] against DENV2, 2.24 [95% CI: 2.21, 2.26] against DENV3 and 1.97 [95% CI: 1.95, 1.99] against DENV4, where the titres below the detection level (<10 1/dil) were assigned a value of 5. 4,251 out of 35,020 subjects (12%) had baseline antibody titres against DENV1-4 quantified by PRNT50 and among the 4,251 subjects 155 (3.6%) were cases. Out of the 30,769 subjects with missing baseline serostatus, 667 (2.2%) had observed PD3 PRNT50 titres, of which 658 (98.6%) were cases.

1.2. Boosted Regression Trees

Technical details on BRT can be found in Elith et al.[4] but intuitively, a BRT model can be thought as a linear combination of trees, where a tree is a partitioning of the predictors space with the objective to identify regions with most homogeneous responses (in our application, the baseline serostatus). BRT models depend on three tuning parameters: (i) tree-complexity (*tc*) that characterises the number of nodes in a tree, (ii) learning rate (*lr*) that determines the contribution of each tree to the growing model (in the example above, it is the multiplicative factor of the trees in the linear combination) and (iii) bag fraction (*bf*) that defines the proportion of data selected for building a tree at each step (*bf* values < 1 introduce randomness in the model, which has been shown to improve the accuracy and reduce overfitting[5]).

Preliminary analyses suggested that BRT models trained and validated on cases and non-cases separately had lower predictive accuracy, especially among cases (sensitivity and specificity around 60%) compared to using cases and non-cases jointly to train the BRT model. This lower performance was due to the relatively small number of cases (155 out of 4,251) in the dataset. For this reason, we built BRT models using the entire dataset, comprising both cases and non-cases, and monitored the model performance overall and on cases and non-cases separately. Using the algorithm specified in section 1.2.2, we tested the effect of different sizes of the training sets and hyper-parameterisations on out-of-sample measures of predictive accuracy.

1.2.1 Optimisation of BRT tuning parameters

We tested 9 different ways of constructing the training the set, resulting from the combination of sampling different proportions (25%, 50% and 75%) of cases and non-cases separately. These scenarios are summarised in Supplementary Table 1.

For each scenario in Supplementary Table 1, we tested 60 different sets of BRT tuning parameters that derived from the combination of five values of tree-complexity (*tc* = 2, 4, 8, 16, 32), four values of the learning rate (*lr* = 0.01, 0.005, 0.001, 0.0005) and three values of the bag fraction (*bf* = 0.5, 0.75, 1). For each of the 540 (= 9 x 60) models defined, we ran 100 random BRT realisations using the algorithm described in section 1.2.2.

Scenario	Percentage (number)	Percentage (number)	Total number
1	25% (39)	25% (1024)	1063
2	25% (39)	50% (2048)	2087
3	25% (39)	75% (3072)	3111
4	50% (78)	25% (1024)	1102
5	50% (78)	50% (2048)	2126
6	50% (78)	75% (3072)	3150
7	75% (116)	25% (1024)	1140
8	75% (116)	50% (2048)	2164
9	75% (116)	75% (3072)	3188

Supplementary Table 1 Percentages and, within parentheses, numbers of cases and non-cases sampled from the 4,251 subjects with observed baseline serostatus to generate the training sets.

1.2.2 Algorithm for tuning parameter optimisation

For given values of *tc, lr* and *bf,* and the proportions of cases and non-cases to be included in the training set, BRT models were built and validated using the following algorithm:

1. Generate the training set by randomly sampling without replacement the specified proportions of cases and non-cases from the subjects with observed baseline serostatus.
2. Generate the out-of-sample validation set, consisting of the subjects with observed baseline serostatus not included in the training set (step 1).
3. Build the BRT model through the gbm.step function in the dismo[6] package, using the training set built in step 1, the specified *tc*, *lr*, *bf*, 10-fold cross-validation and increasing the model in steps of 50 trees at each iteration, using the baseline serostatus (seronegative/seropositive) as response variable and the PD3 PRNT50 titres, trial, country, age, gender, case occurrence, the infecting serotype and the time interval between dose 3 and symptoms onset as predictors. The optimal number of trees minimising the holdout (cross-validation) deviance is computed.
4. Use the BRT model built in step 3 to predict the baseline serostatus of the subjects in the validation set. For each subject i in the validation set, the BRT model gives the probability p_i that subject i is seropositive.
5. Define the cut-off threshold p_T, by which subjects with $p_i > p_T$ are classified as seropositive or seronegative otherwise. We defined a separate optimal cut-off threshold for cases and non-cases, having chosen cut-off values giving equal sensitivity and specificity using the optimal.threshold function in the PresenceAbsence[7] package.
6. Use the cut-off threshold p_T computed in step 5 to classify the cases and non-cases in the validation set as seronegative or seropositive.
7. Compute the sensitivity (proportion of baseline seropositive subjects correctly classified), specificity (proportion of baseline seronegative subjects correctly classified) and percentage of predictions correctly classified in the validation set overall and among cases and non-cases separately.

The choice of optimising the cut-off thresholds to have equal sensitivity and specificity in step 5 was made to avoid introducing bias in data imputation.

In a sensitivity analysis, we tested the effect of using a single cut-off threshold p_T computed over all subjects (i.e. cases and non-case) in step 5. Despite the cut-off threshold being chosen to give equal sensitivity and specificity over all subjects, the relatively small number of cases in the samples resulted in 14% lower sensitivity than specificity for the cases.

1.2.3 Model simplification

Using the gbm.simplify function of the dismo[6] package, we tested the effect of removing up to 9 variables from the model. Using 10-fold cross-validation, this function computes the change in holdout deviance obtained by removing an increasing number of variables (from the lowest to the highest contributing predictors) and returns the variables which removal improves the holdout deviance compared to the full model. We ran the model simplification on 100 realisations of the BRT model, each one trained on a randomly generated training set (built by sampling 50% of the non-cases and 75% of the cases) using the optimal parameterisation (*tc* = 16, *lr* = 0.0005 and *bf* = 0.75). In Supplementary Table 2 we present the results of the model simplification in terms of percentage of variables dropped in the 100 random BRT realisations.

1.2.4 Vaccine efficacy computation

Denote

N = number of subjects with observed baseline serostatus

C = number of cases with observed baseline serostatus

$\widetilde{N}$= number of subjects with missing baseline serostatus and observed PD3 titres against DENV1-4, i.e. with imputed baseline serostatus

$\tilde{C}$= number of cases with missing baseline serostatus and observed PD3 titres against DENV1-4, i.e. with imputed baseline serostatus

$\widehat{N}$= number of subjects with missing baseline serostatus and missing PD3 titres against DENV1-4

$\hat{C}$= number of cases with missing baseline serostatus and missing PD3 titres against DENV1-4

Superscripts V, C refer to the vaccine and control group respectively, and superscripts $V1$, $V0$ denote subjects in the vaccine group who were baseline seropositive or seronegative respectively. Similarly, superscripts $C1$, $C0$ denote the subjects in the control group who were baseline seropositive or seronegative, respectively.

We use the observed and imputed baseline serostatus of subjects in the vaccine and control arms to infer the number of individuals with missing PD3 PRNT50 titres who were seropositive or seronegative at baseline, using the following equations:

$$\hat{C}^{V1} = \hat{C}^{V}\left(\frac{C^{V1}+\tilde{C}^{V1}}{C^{V}+\tilde{C}^{V}}\right) \quad (1)$$

$$\hat{C}^{V0} = \hat{C}^{V} - \hat{C}^{V1} = \hat{C}^{V}\left(\frac{C^{V0}+\tilde{C}^{V0}}{C^{V}+\tilde{C}^{V}}\right) \quad (2)$$

$$\widehat{N}^{V1} = \widehat{N}^{V}\left(\frac{N^{V1}+\widetilde{N}^{V1}}{N^{V}+\widetilde{N}^{V}}\right) \quad (3)$$

$$\widehat{N}^{V0} = \widehat{N}^{V} - \widehat{N}^{V1} = \widehat{N}^{V}\left(\frac{N^{V0}+\widetilde{N}^{V0}}{N^{V}+\widetilde{N}^{V}}\right) \quad (4)$$

$$\hat{C}^{C1} = \hat{C}^{C}\left(\frac{C^{C1}+\tilde{C}^{C1}}{C^{C}+\tilde{C}^{C}}\right) \quad (5)$$

$$\hat{C}^{C0} = \hat{C}^{C} - \hat{C}^{C1} = \hat{C}^{C}\left(\frac{C^{C0}+\tilde{C}^{C0}}{C^{C}+\tilde{C}^{C}}\right) \quad (6)$$

$$\widehat{N}^{C1} = \widehat{N}^{C}\left(\frac{N^{C1} + \widetilde{N}^{C1}}{N^{C} + \widetilde{N}^{C}}\right) \quad (7)$$

$$\widehat{N}^{C0} = \widehat{N}^{C} - \widehat{N}^{C1} = \widehat{N}^{C}\left(\frac{N^{C0} + \widetilde{N}^{C0}}{N^{C} + \widetilde{N}^{C}}\right) \quad (8)$$

Using equations (1) – (8), the attack rates in the vaccine and control arms in the baseline seropositive and seronegative groups can be expressed as:

$$AR^{V1} = \frac{C^{V1} + \tilde{C}^{V1} + \hat{C}^{V1}}{N^{V1} + \widetilde{N}^{V1} + \widehat{N}^{V1}} = \frac{\left(C^{V1} + \tilde{C}^{V1}\right)\frac{(C^{V} + \tilde{C}^{V} + \hat{C}^{V})}{(C^{V} + \tilde{C}^{V})}}{\left(N^{V1} + \widetilde{N}^{V1}\right)\frac{(N^{V} + \widetilde{N}^{V} + \widehat{N}^{V})}{(N^{V} + \widetilde{N}^{V})}} \quad (9)$$

$$AR^{C1} = \frac{C^{C1} + \tilde{C}^{C1} + \hat{C}^{C1}}{N^{C1} + \widetilde{N}^{C1} + \widehat{N}^{C1}} = \frac{\left(C^{C1} + \tilde{C}^{C1}\right)\frac{(C^{C} + \tilde{C}^{C} + \hat{C}^{C})}{(C^{C} + \tilde{C}^{C})}}{\left(N^{C1} + \widetilde{N}^{C1}\right)\frac{(N^{C} + \widetilde{N}^{C} + \widehat{N}^{C})}{(N^{C} + \widetilde{N}^{C})}} \quad (10)$$

$$AR^{V0} = \frac{C^{V0} + \tilde{C}^{V0} + \hat{C}^{V0}}{N^{V0} + \widetilde{N}^{V0} + \widehat{N}^{V0}} = \frac{\left(C^{V0} + \tilde{C}^{V0}\right)\frac{(C^{V} + \tilde{C}^{V} + \hat{C}^{V})}{(C^{V} + \tilde{C}^{V})}}{\left(N^{V0} + \widetilde{N}^{V0}\right)\frac{(N^{V} + \widetilde{N}^{V} + \widehat{N}^{V})}{(N^{V} + \widetilde{N}^{V})}} \quad (11)$$

$$AR^{C0} = \frac{C^{C0} + \tilde{C}^{C0} + \hat{C}^{C0}}{N^{C0} + \widetilde{N}^{C0} + \widehat{N}^{C0}} = \frac{\left(C^{C0} + \tilde{C}^{C0}\right)\frac{(C^{C} + \tilde{C}^{C} + \hat{C}^{C})}{(C^{C} + \tilde{C}^{C})}}{\left(N^{C0} + \widetilde{N}^{C0}\right)\frac{(N^{C} + \widetilde{N}^{C} + \widehat{N}^{C})}{(N^{C} + \widetilde{N}^{C})}} \quad (12)$$

Vaccine efficacy estimates with imputed data (including group-level inference of the baseline serostatus) among baseline seropositive (VE^1) and seronegative (VE^0) subjects are given by:

$$VE^{1} = 1 - \frac{AR^{V1}}{AR^{C1}} \quad (13)$$

$$VE^{0} = 1 - \frac{AR^{V0}}{AR^{C0}} \quad (14)$$

Vaccine efficacy estimates with imputed data by baseline serostatus and trial, age and country are computed using equations (13) – (14) on the data stratified by trial, age and country, respectively.

Vaccine efficacy estimates with imputed data by baseline serostatus and serotype j ($j = 1, \ldots, 4$) are computed using equations (13) – (14) with the infecting serotype j specified for the cases. Let for instance C_j^V and C_j^C represent the number of cases with observed baseline serostatus infected by serotype j in the vaccine and control group, respectively. The vaccine efficacy against serotype j among baseline seropositive subjects is given by:

$$VE_j^1 = 1 - \frac{AR_j^{V1}}{AR_j^{C1}} \tag{15}$$

where

$$AR_j^{V1} = \frac{\left(C_j^{V1} + \tilde{C}_j^{V1}\right)\frac{(C_j^V + \tilde{C}_j^V + \hat{C}_j^V)}{(C_j^V + \tilde{C}_j^V)}}{\left(N^{V1} + \widetilde{N}^{V1}\right)\frac{(N^V + \widetilde{N}^V + \widehat{N}^V)}{(N^V + \widetilde{N}^V)}} \tag{16}$$

$$AR_j^{C1} = \frac{\left(C_j^{C1} + \tilde{C}_j^{C1}\right)\frac{(C_j^C + \tilde{C}_j^C + \hat{C}_j^C)}{(C_j^C + \tilde{C}_j^C)}}{\left(N^{C1} + \widetilde{N}^{C1}\right)\frac{(N^C + \widetilde{N}^C + \widehat{N}^C)}{(N^C + \widetilde{N}^C)}} \tag{17}$$

Vaccine efficacy estimates including individual-level imputations only (i.e. without the group-level inference of baseline serostatus) are given by equations (13) and (14), having set $\hat{N}$ and $\hat{C}$ in equations (9) – (12) equal to zero.

Vaccine efficacy estimates without imputed data are calculated using equations (13) and (14), having set $\tilde{N}$, $\tilde{C}$, $\hat{N}$, $\hat{C}$ in equations (9) – (12) equal to zero (i.e. the attack rates in this case are given by the observed proportion of cases in each group).

1.2.5 Algorithm for vaccine efficacy estimation

1.2.5.1 Mean estimate

The mean vaccine efficacy estimates with imputation were calculated from 1,000 realisations of steps 1 – 7 in Supplementary section 1.2.2, followed by steps 8 and 9 below:

8. Use the BRT model built in step 4 to impute the baseline serostatus of the subjects with missing baseline serostatus and observed PD3 PRNT50 titres
9. Calculate the vaccine efficacy using the subjects receiving 3 vaccine doses (per-protocol population) as described in Supplementary section 1.2.4

1.2.5.2 Confidence interval

Confidence intervals around the mean vaccine efficacy estimates were calculated by bootstrapping. Using the optimal tuning parameter set (*tc* = 16, *lr* = 0.0005, *bf* = 0.75), the 95% CI of the vaccine efficacy estimates with imputation were calculated as the 2.5-97.5 percentiles of 1,000 random realisations of the algorithm detailed below (steps 2 – 9 below are the same as steps 1 – 7 in Supplementary section 1.2.2):

1. Generate a synthetic population by sampling with replacement the 35,020 subjects enrolled in the trials.
2. Generate the training set by sampling without replacement 50% of cases and 75% of non-cases from the synthetic population sampled in step 1 with complete baseline serostatus

3. Generate the out-of-sample validation set – i.e. the subjects in the synthetic population generated in step 1 with complete baseline serostatus who are not included in the training set (step 2)
4. Build the BRT model through the gbm.step function in the dismo[7] package, using the training set build in step 3, *tc* = 16, *lr* = 0.0005, *bf* = 0.75, 10-fold cross-validation and increasing the model in steps of 50 trees at each iteration, using the baseline serostatus (seronegative/seropositive) as response variable and the PD3 PRNT50 titres, country, age, gender, the infecting serotype and the time interval between dose 3 and symptoms onset as predictors.
5. Use the BRT model built in step 4 to predict the baseline serostatus of the subjects in the validation set. For each subject i in the validation set, the BRT model calculates the probability p_i that subject i is seropositive.
6. Define the cut-off threshold p_T, by which subjects with $p_i > p_T$ are classified as seropositive or seronegative otherwise. We defined a separate cut-off threshold for cases and non-cases separately, having chosen cut-off values giving equal sensitivity and specificity using the optimal.threshold function in the PresenceAbsence[5] package.
7. Use the cut-off thresholds p_T computed in step 6 to classify the cases and non-cases in the validation set generated in step 3 as seronegative or seropositive.
8. Compute the sensitivity (proportion of baseline seropositive subjects correctly classified), specificity (proportion of baseline seronegative subjects correctly classified) and percentage of predictions correctly classified in the validation set overall and among cases and non-cases separately.
9. Use the BRT model built in step 4 to impute the baseline serostatus of the subjects in the synthetic prediction set (i.e. the subjects sampled in step 1 with missing baseline serostatus and observed PD3 PRNT50 titres)
10. Calculate the seroprevalence among the subjects with complete and imputed baseline serostatus
11. Calculate the vaccine efficacy using the subjects receiving 3 vaccine doses (per-protocol population) as described in Supplementary section 1.2.4

1.2.6. Algorithm to estimate the amount of variation due to incomplete baseline serostatus

We quantified the percentage of variance due to incomplete baseline serostatus among the subjects with observed PD3 titres as detailed below:

1. Calculate the variance of the vaccine efficacy estimates obtained from the realisations obtained in Supplementary section 1.2.5.2 (these are the realisations used to calculate the 95% CI). Call this variance F.
2. For 1,000 times, repeat
   a. steps 1 – 9 described in Supplementary section 1.2.5.1 followed by
   b. step 10 – for 100 times do
      i. sample with replacement the 35,020 subjects enrolled in the trials
      ii. calculate the vaccine efficacy for the set obtained at step i. above
   c. Calculate the variance of the vaccine efficacy estimates obtained at step ii. Call this variance C (note that we have 1,000 estimates of C, one for each realisation of step 2).
3. Calculate the mean (out of the 1,000 realisations) of C and call it $\bar{C}$
4. The relative increase in the variance of vaccine efficacy due to incomplete baseline samples among the subjects with observed PD3 titres is given by $(F - \bar{C})/\bar{C}$

2. Supplementary Figures and Tables

2.1. Tuning parameter and training set size optimisation

Supplementary Figures 1 – 9 show the sensitivity, specificity and percentage of predictions correctly classified among out-of-sample cases and non-cases separately and overall, using a grid search on the tuning parameters, as described in Supplementary section 1.2.1. Following Elith et al.[4], we only retained models with at least 1,000 trees.

To avoid imputations introducing bias into the data we optimised two separate cut-off thresholds used for classification, one for cases and one for non-cases (separately), so that imputation returned the same sensitivities and specificities[7]. This implies that the proportion of predictions correctly classified for cases and non-cases (separately) is also equal to the sensitivity/specificity of the cases and non-cases (separately).

The optimal parameterisation was selected using the following steps:

1. Calculate the highest lower bound of the confidence interval of sensitivity/specificity /proportion predictions correctly classified among cases.
2. Select the parameterisations that produce statistically equivalent sensitivity/specificity/ proportion predictions correctly classified among cases. This is obtained by selecting the parameterisations that have the upper bound of the confidence interval of sensitivity/specificity/proportion predictions correctly classified among cases larger or equal than the indicator obtained in step 1.
3. Among the parameterisations selected in step 2, choose the one giving the highest upper bound of the confidence interval of the sensitivity/specificity/proportion of predictions correctly classified among non-cases.

We preferred to prioritise the maximisation of the accuracy measures among cases because imputation was mainly conducted on cases due to the trial design.

Steps 1-3 returned the parameterisation adopted in the final BRT model, i.e. tree complexity (*tc*) = 16, learning rate (*lr*) = 0.0005, bag fraction (*bf*) = 0.75 and training sets composed by 75% of cases and 50% of non-cases with complete information on the baseline serostatus. As a sensitivity analysis we compared the accuracy obtained with grid search with the accuracy obtained using random search. Supplementary Figure 10 shows the optimal accuracy obtained with random search, which was conducted by drawing 1,000 random parameterisations of *tc* (integer distribution between 2 and 50), *lr* (uniform distribution between 0.0005 and 0.01), *bf* (uniform distribution between 0.5 and 1) and random numbers of cases and non-cases with complete information on the baseline serostatus (integer distribution between 39 and 116 for cases and between 1,024 and 3,072 for non-cases). The optimal parameterisation obtained in the random search had *tc* = 43, *lr* =0.002 , *bf* = 0.71 and training sets composed by 59% (91/155) of cases and 66% (2,729/4,096) of non-cases with complete information on the baseline serostatus. Supplementary Figure 10 shows that the accuracy obtained with this parameterisation is equivalent to the accuracy obtained with the optimal parameterisation obtained with grid search. The robustness of the accuracy achieved by BRT to the choice of the hyper-parameterisation is due to the fact that in BRT the number of trees grown is optimised according to the hyper-parameterisation used (this is markedly different from random forest for instance, where the total number of trees grown is given as a parameter). In this regard, random search identified a hyper-parametrisation that achieved the same accuracy obtained with grid search but using less computational time (i.e. requiring a smaller number of trees to be grown).

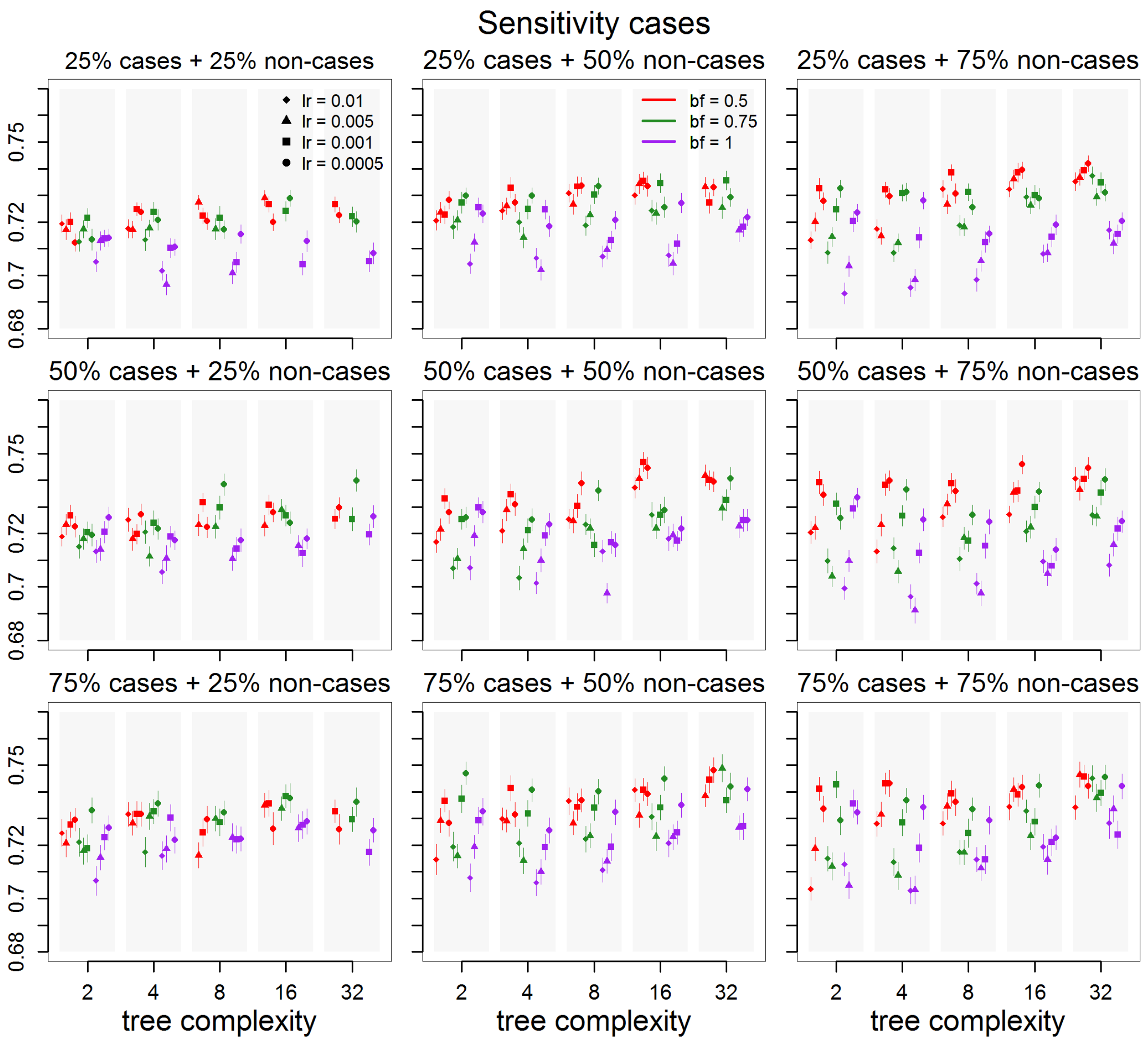

Supplementary Figure 1 Out-of-sample sensitivity (proportion of baseline seropositive subjects correctly classified) among cases (mean and 95% CI) using all combinations of tree complexities (tc = 2, 4, 8, 16, 32), learning rates (lr = 0.01, 0.005, 0.001, 0.0005), bag fractions (bf = 0.5, 0.75, 1) and training sets (Supplementary Table 1) from 100 randomly sampled training and validation sets (Supplementary section 1.2.2). Only BRT models with at least 1,000 trees are shown.

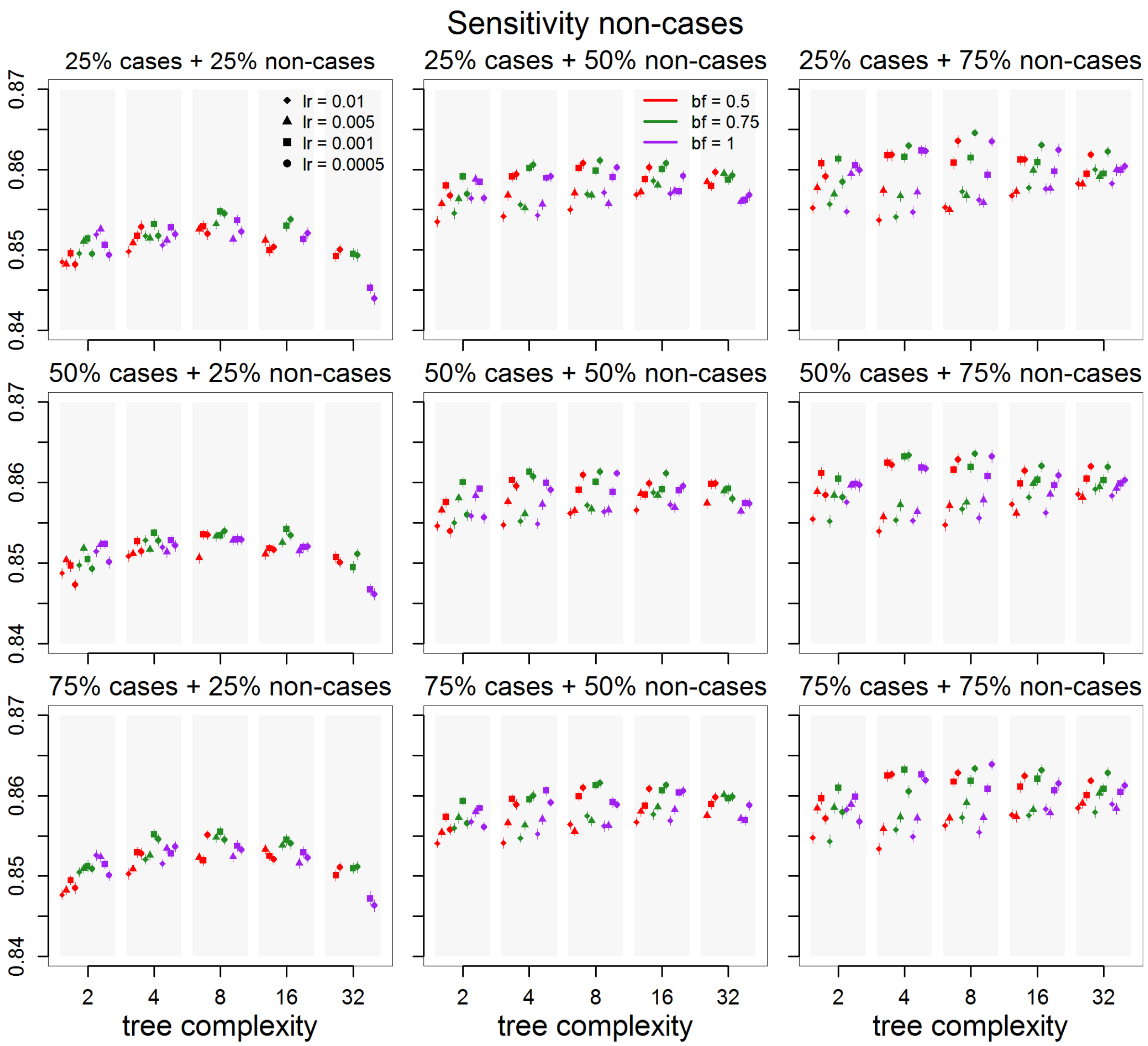

Supplementary Figure 2 Out-of-sample sensitivity (proportion of baseline seropositive subjects correctly classified) among non-cases (mean and 95% CI) using all combinations of tree complexities (tc = 2, 4, 8, 16, 32), learning rates (lr = 0.01, 0.005, 0.001, 0.0005), bag fractions (bf = 0.5, 0.75, 1) and training sets (Supplementary Table 1) from 100 randomly sampled training and validation sets (Supplementary section 1.2.2). Only BRT models with at least 1,000 trees are shown.

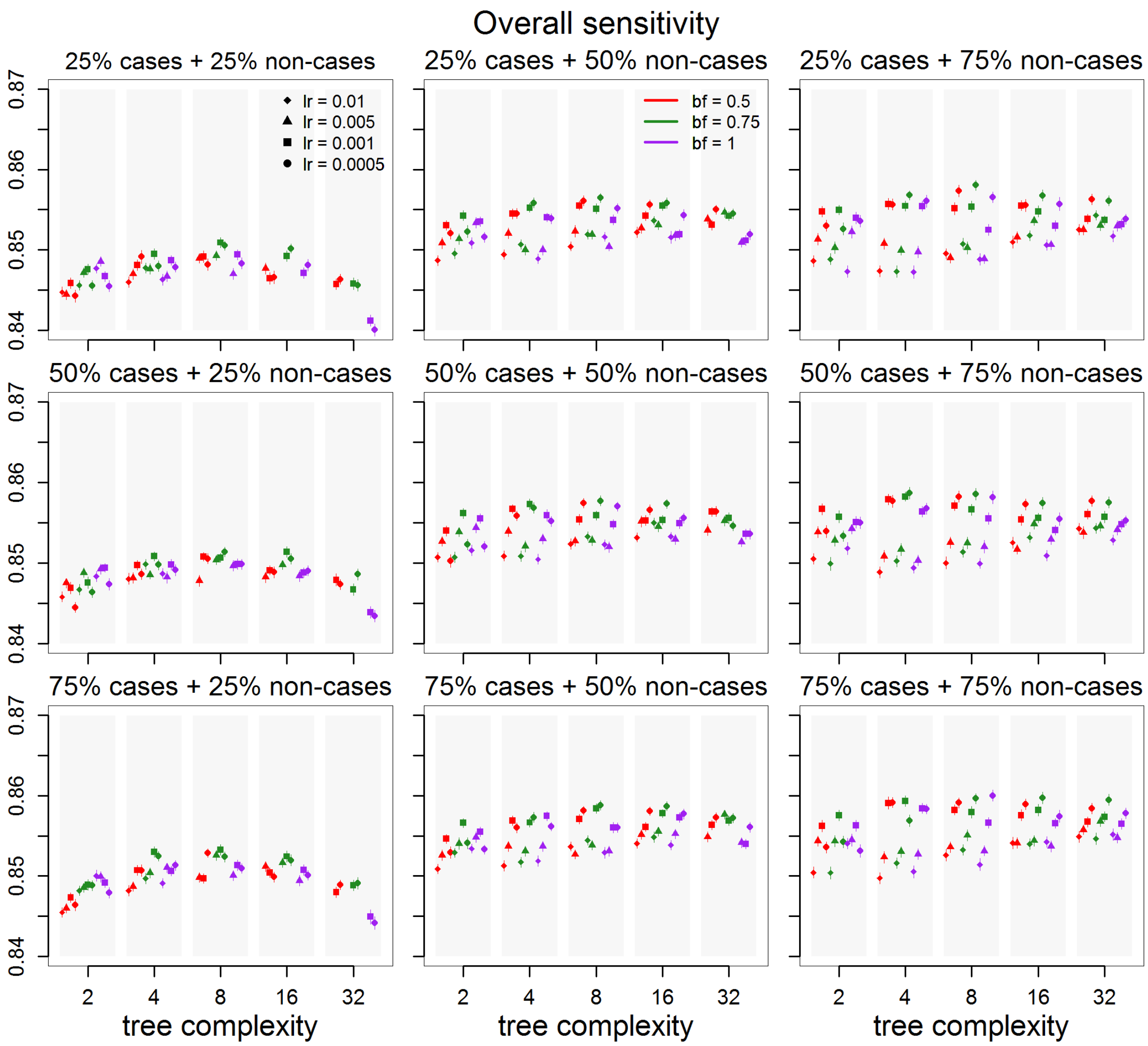

Supplementary Figure 3 Out-of-sample sensitivity (proportion of baseline seropositive subjects correctly classified) overall (mean and 95% CI) using all combinations of tree complexities (tc = 2, 4, 8, 16, 32), learning rates (lr = 0.01, 0.005, 0.001, 0.0005), bag fractions (bf = 0.5, 0.75, 1) and training sets (Supplementary Table 1) from 100 randomly sampled training and validation sets (Supplementary section 1.2.2). Only BRT models with at least 1,000 trees are shown.

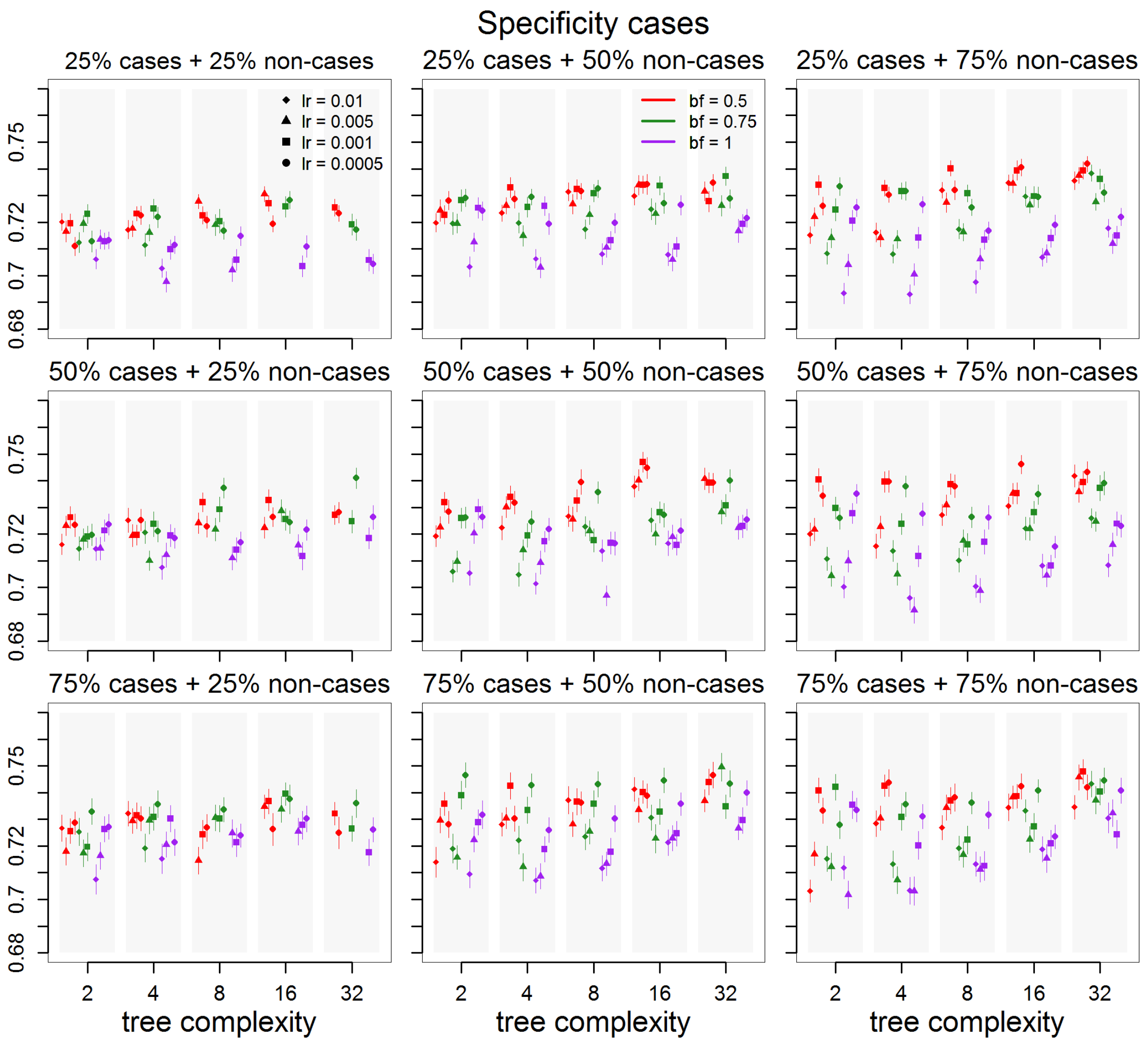

Supplementary Figure 4 Out-of-sample specificity (proportion of baseline seronegative subjects correctly classified) among cases (mean and 95% CI) using all combinations of tree complexities (tc = 2, 4, 8, 16, 32), learning rates (lr = 0.01, 0.005, 0.001, 0.0005), bag fractions (bf = 0.5, 0.75, 1) and training sets (Supplementary Table 1) from 100 randomly sampled training and validation sets (Supplementary section 1.2.2). Only BRT models with at least 1,000 trees are shown.

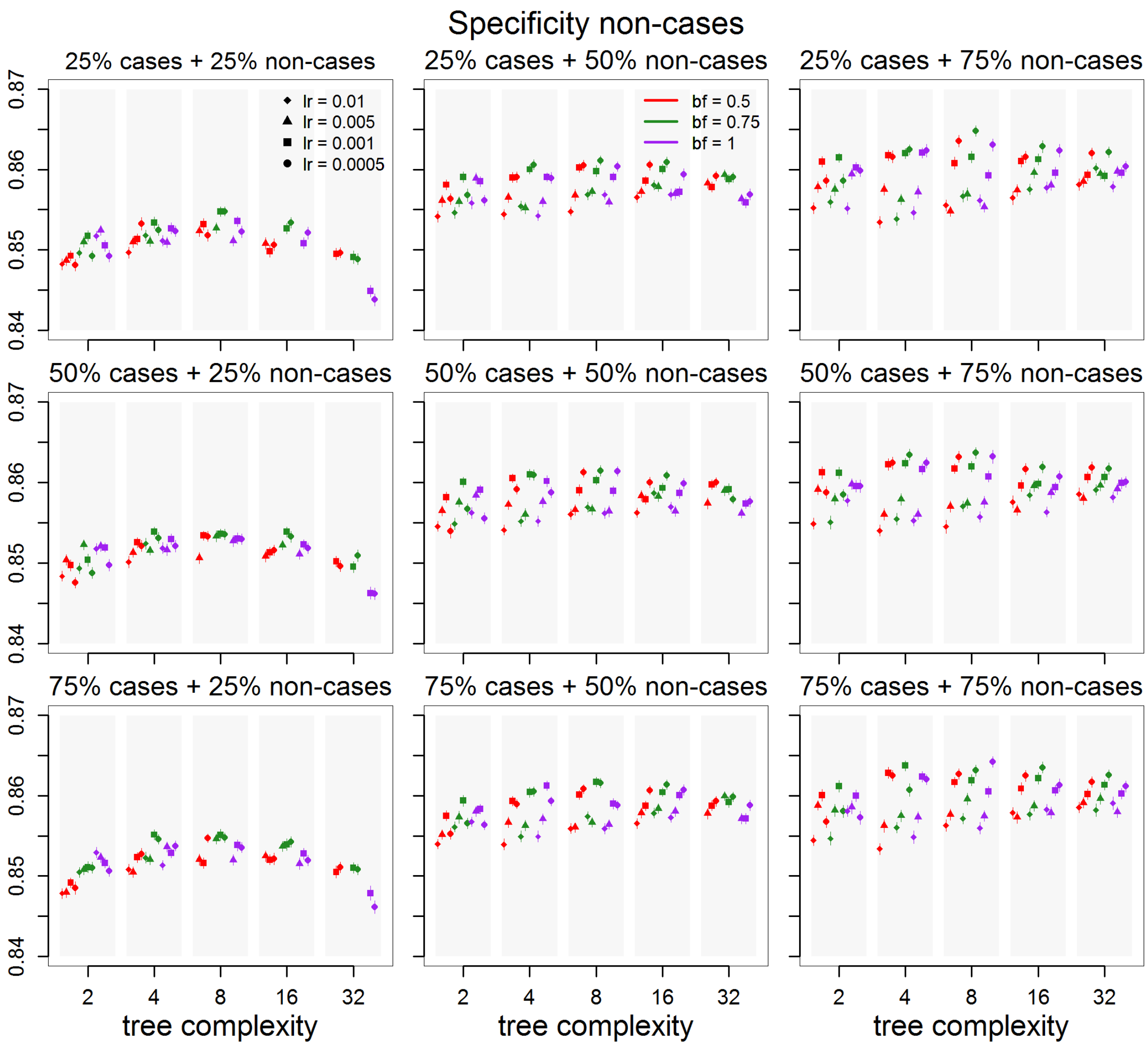

Supplementary Figure 5 Out-of-sample specificity (proportion of baseline seronegative subjects correctly classified) among non-cases (mean and 95% CI) using all combinations of tree complexities (tc = 2, 4, 8, 16, 32), learning rates (lr = 0.01, 0.005, 0.001, 0.0005), bag fractions (bf = 0.5, 0.75, 1) and training sets (Supplementary Table 1) from 100 randomly sampled training and validation sets (Supplementary section 1.2.2). Only BRT models with at least 1,000 trees are shown.

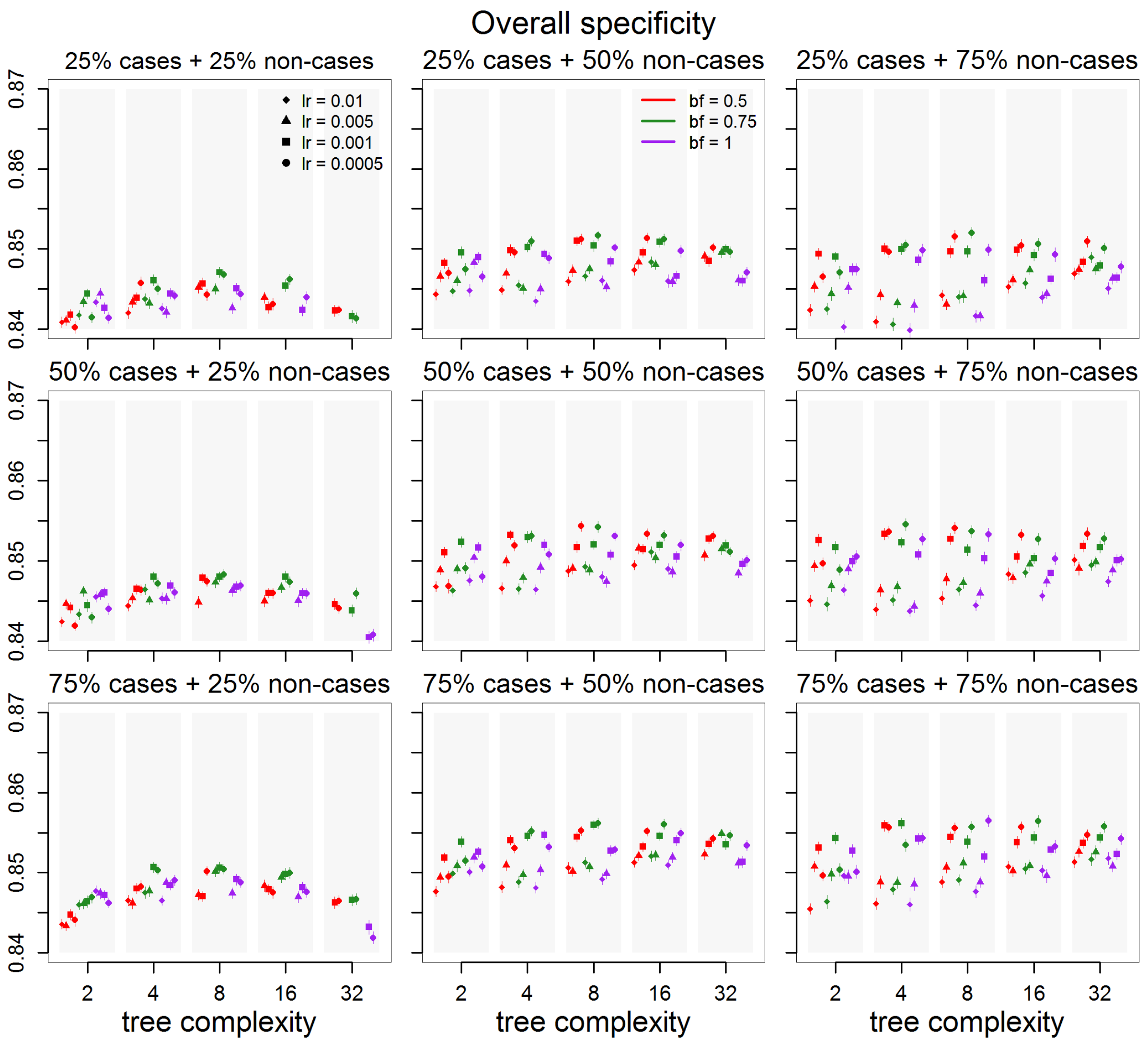

Supplementary Figure 6 Out-of-sample specificity (proportion of baseline seronegative subjects correctly classified) overall (mean and 95% CI) using all combinations of tree complexities (tc = 2, 4, 8, 16, 32), learning rates (lr = 0.01, 0.005, 0.001, 0.0005), bag fractions (bf = 0.5, 0.75, 1) and training sets (Supplementary Table 1) from 100 randomly sampled training and validation sets (Supplementary section 1.2.2). Only BRT models with at least 1,000 trees are shown.

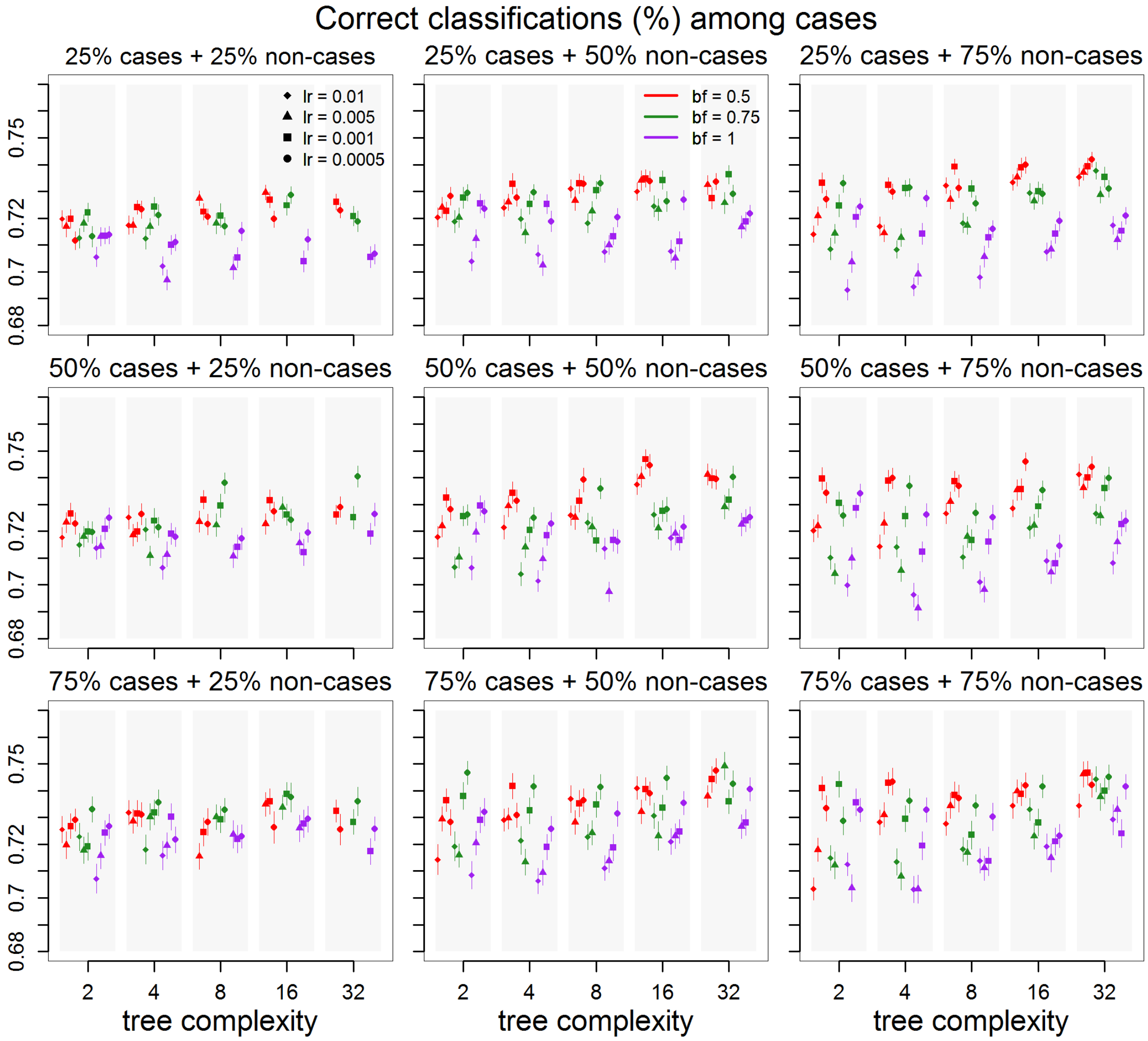

Supplementary Figure 7 Out-of-sample proportion of correct classifications (probability of correctly classifying the baseline serostatus) among cases (mean and 95% CI) using all combinations of tree complexities (tc = 2, 4, 8, 16, 32), learning rates (lr = 0.01, 0.005, 0.001, 0.0005), bag fractions (bf = 0.5, 0.75, 1) and training sets (Supplementary Table 1) from 100 randomly sampled training and validation sets (Supplementary section 1.2.2). Only BRT models with at least 1,000 trees are shown.

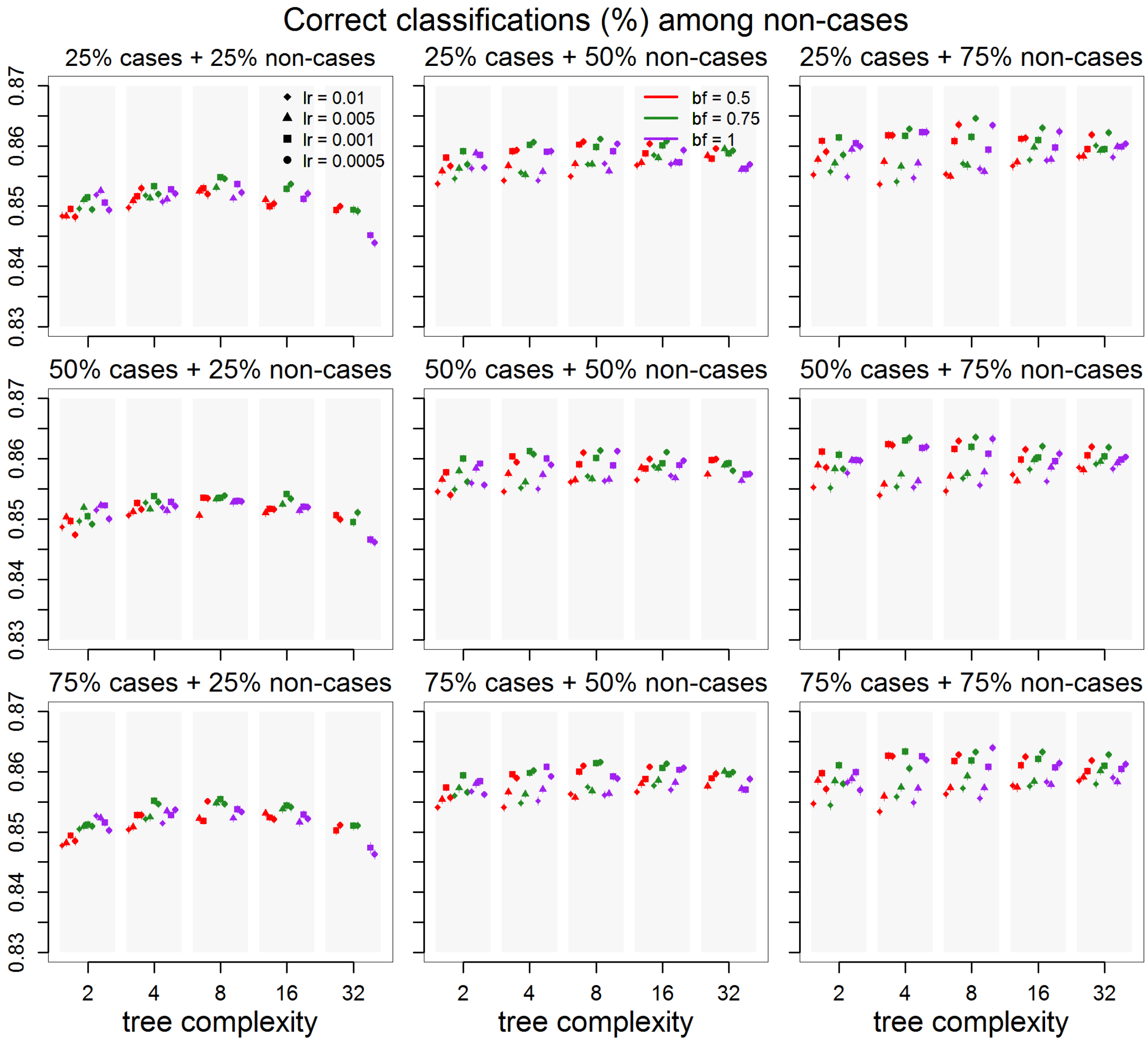

Supplementary Figure 8 Out-of-sample proportion of correct classifications (probability of correctly classifying the baseline serostatus) among non-cases (mean and 95% CI) using all combinations of tree complexities (tc = 2, 4, 8, 16, 32), learning rates (lr = 0.01, 0.005, 0.001, 0.0005), bag fractions (bf = 0.5, 0.75, 1) and training sets (Supplementary Table 1) from 100 randomly sampled training and validation sets (Supplementary section 1.2.2). Only BRT models with at least 1,000 trees are shown.

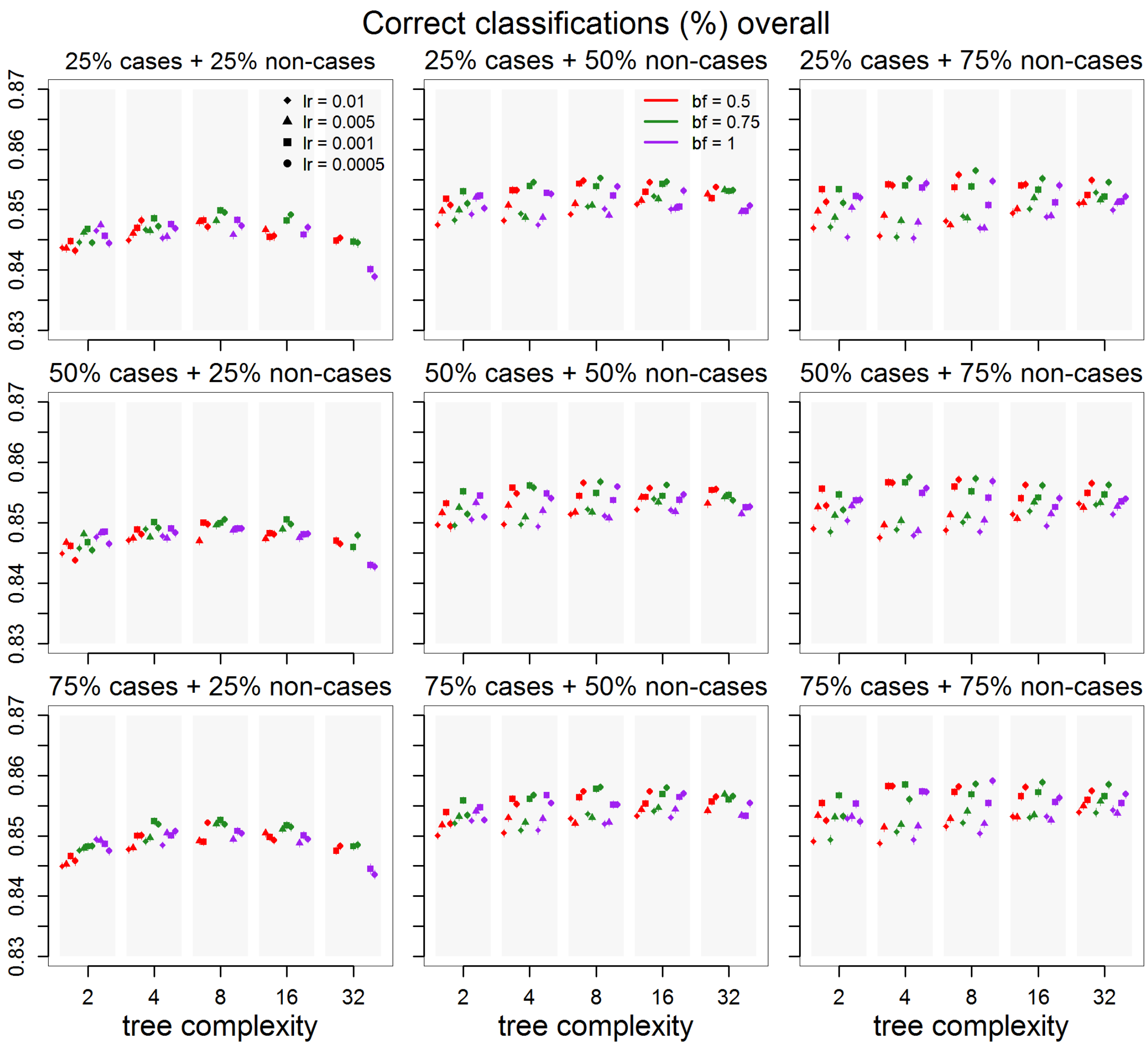

Supplementary Figure 9 Out-of-sample proportion of correct classifications (probability of correctly classifying the baseline serostatus) overall (mean and 95% CI) using all combinations of tree complexities (tc = 2, 4, 8, 16, 32), learning rates (lr = 0.01, 0.005, 0.001, 0.0005), bag fractions (bf = 0.5, 0.75, 1) and training sets (Supplementary Table 1) from 100 randomly sampled training and validation sets (Supplementary section 1.2.2). Only BRT models with at least 1,000 trees are shown.

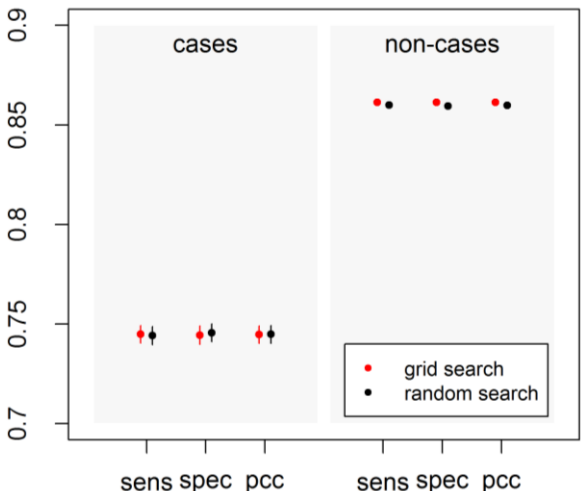

Supplementary Figure 10 Out-of-sample sensitivity, specificity and proportion of correct classifications (probability of correctly classifying the baseline serostatus) among cases and non-cases (mean and 95% CI) using the optimal parametrisation found using grid search (tc = 16, lr = 0.0005, bf = 0.75 and training sets composed by 75% of cases and 50% of non-cases with complete information on the baseline serostatus) and random search (tc = 43, lr = 0.002, bf = 0.71 and training set composed by 59% (91/155) of cases and 66% (2,729/4,096) of non-cases with complete information on the baseline serostatus).

2.2 Model simplification

We found that trial, case, gender and serotype were dropped in the 65%, 55%, 21% and 18% of the realisations respectively, while all other predictors (PD3 PRNT50 titres, country, arm, age and time interval between dose 3 and symptoms onset) were always retained into the model. The final (simplified) BRT model used all variables (PD3 PRNT50 titres, country, arm, serotype, age, time interval between dose 3 and symptoms onset and gender) except trial and case as predictors.

Supplementary Table 2 summarises the relative importance of the variables included in the full and final models. The relative contributions have been averaged over 100 realisations of BRT models trained on randomly sampled training sets including 50% of the non-cases and 75% of the cases and using *tc* = 16, *lr* = 0.0005 and *bf* = 0.75.

Predictors	Full model	Final model
PD3 PRNT50 titre against DENV1	29.2	29.4
PD3 PRNT50 titre against DENV2	20.3	20.8
Country	11.9	12.0
PD3 PRNT50 titre against DENV3	11.5	11.3
Age	10.3	10.3
PD3 PRNT50 titre against DENV4	7.6	7.7
Arm	5.1	5.0
Serotype	1.5	1.5
Time interval between dose 3 to symptoms	1.4	1.3
Gender	0.7	0.8
Trial	0.3	-
Case	0.1	-

Supplementary Table 2 Relative contributions (%) of the predictors used in BRT models using 10-fold cross-validation, tc = 16, lr = 0.0005 and bf = 0.75, and trained on 100 training sets generated by randomly sampling 50% of non-cases and 75% of cases with observed baseline serostatus. The full model used all 12 predictors and the final model used 10 predictors.

Supplementary Figure 11A shows the accuracy obtained with the full model and the final model. In a sensitivity analysis we tested the accuracy of a more parsimonious (minimal) model that used the predictors with relative contributions > 1.5 only (Supplementary Table 2), i.e. with gender, time interval between dose 3 and symptoms onset and serotype removed. The small yet visible drop in average performance of the minimal model compared to the full model (Supplementary Figure 11B) indicated the adequacy of the use of the final (as opposed to the minimal) model for data imputation.

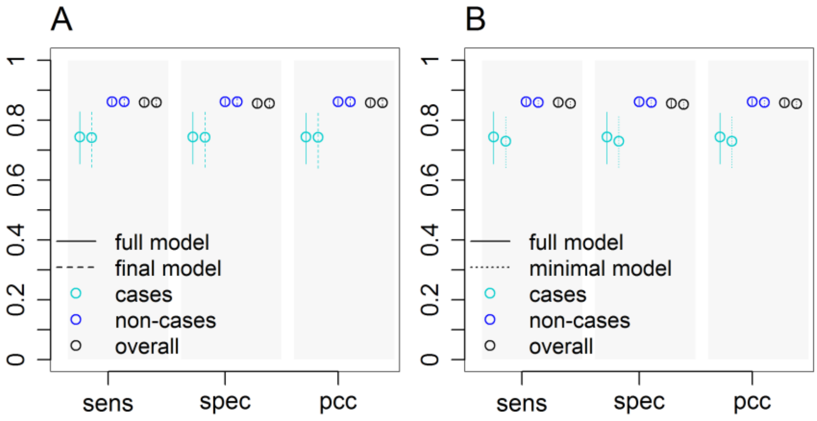

Supplementary Figure 11 Mean and 95% CI of the out-of-sample sensitivity (sens), specificity (spec) and percentage of predictions correctly classified (pcc) computed from 100 random realisation of BRT models developed with 10-fold cross-validation, using tc = 16, lr = 0.0005 and bf = 0.75 and trained on 50% of non-cases and 75% of cases in the immunogenicity subset. (A) Comparison between the full model (solid lines) and the final model (dashed lines); (B) comparison between the full model (solid lines) and the minimal model (dotted lines).

2.3 Estimated seroprevalence using the final BRT model

Supplementary Figure 12 shows a comparison of the observed and estimated seroprevalence among cases (first row) and non-cases (second row) by country (first column) and by country and age-group (second column). The 95% CI around the observed seroprevalence was obtained by bootstrapping. The mean and 95% CI of the estimated seroprevalence were calculated over 1,000 realisations of the final BRT model, having included sampling variability (as discussed in Supplementary section 1.2.5) in each realisation.

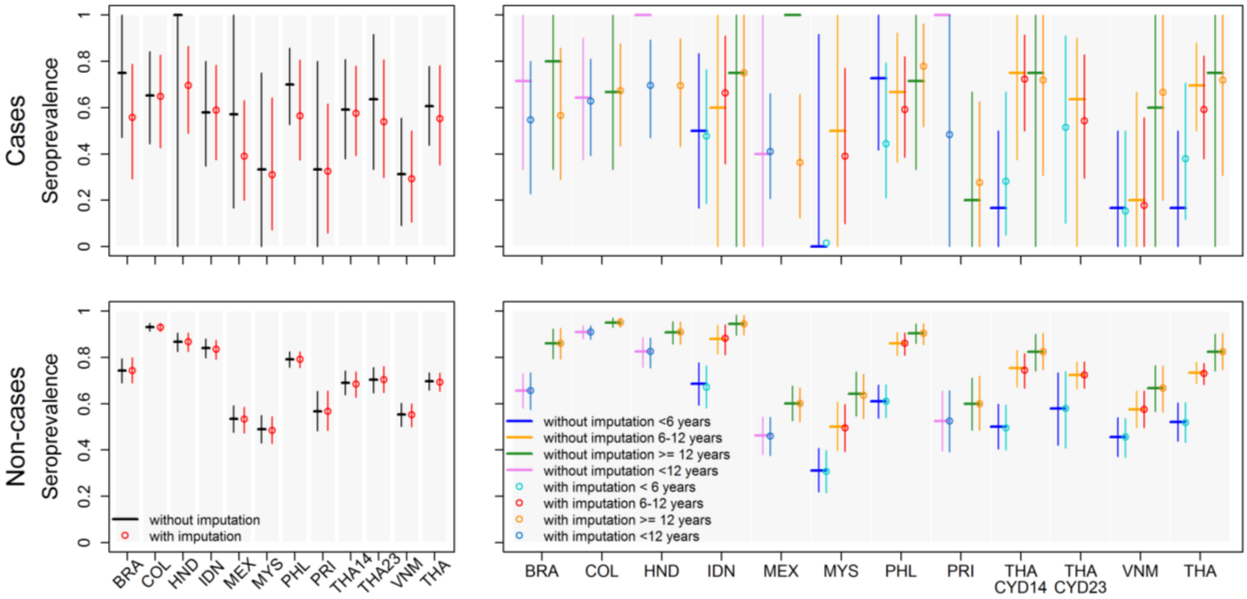

Supplementary Figure 12 Mean and 95% CI of the observed and estimated seroprevalence among cases (first row) and non-cases (second row) by country (first column) and by country and age-group (second column). Estimates were obtained from 1,000 realisations of the final BRT model trained on 50% of non-cases and 75% of cases and using 10-fold cross-validation, tc = 16, lr = 0.0005, bf = 0.75.

2.4 Estimated vaccine efficacy using the final model, additional results

Supplementary Figure 13 shows the observed and estimates vaccine efficacy by baseline serostatus and country, trial, serotype and age for South-east Asia and Latin America separately. These estimates were obtained using the final BRT model and the optimal hyper-parameterisation (*tc* = 16, *lr* = 0.0005 and *bf* = 0.75, with training sets generated by sampling 50% of the non-cases and 75% of the cases), from the same 1,000 realisations that generated the results in Figure 1 in the main text.

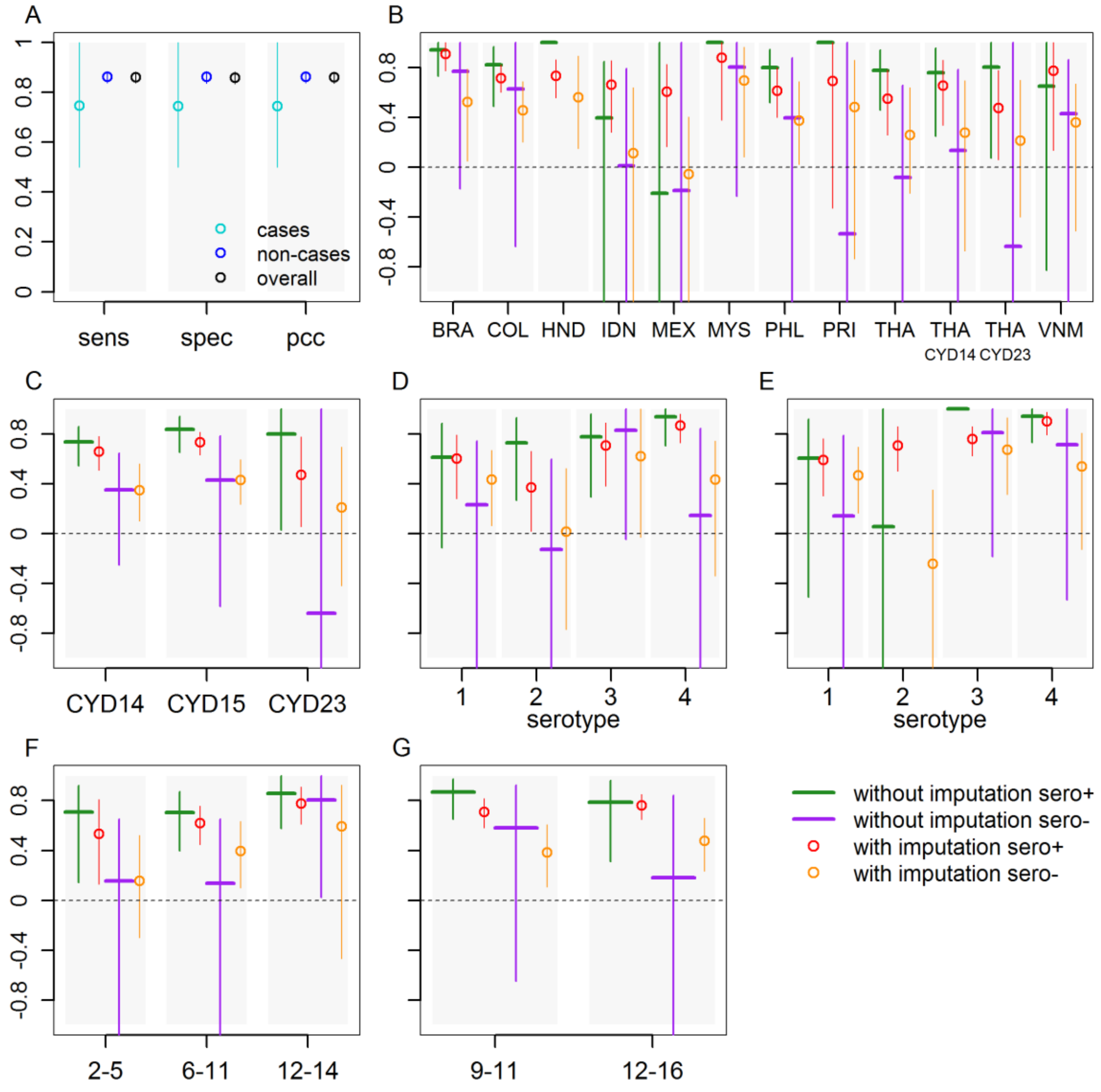

Supplementary Figure 13 Mean and 95% CI of the vaccine efficacy estimates generated with and without imputed data for baseline seronegative (sero-) and baseline seropositive (sero+) subjects. Estimates were obtained from 1,000 realisations of the final BRT model trained on 50% of non-cases and 75% of cases and using 10-fold cross-validation, tc = 16, lr = 0.0005, bf = 0.75. A) sensitivity (sens), specificity (spec) and proportion of correct classifications (pcc) among cases, non-cases and overall; B) vaccine efficacy estimates by country for baseline seropositive and baseline seronegative subjects (BRA = Brazil, OL = Colombia, HND = Honduras, IDN = Indonesia, MEX = Mexico, MYS = Malaysia, PHL = Philippines, PRI = Puerto Rico, THA = Thailand, VNM = Vietnam). Efficacy estimates for Thailand were generated using the phse-2b (CYD23) and the phase-3 (CYD14) data separately and combined; C) vaccine efficacy estimates by trial (CYD14 = phase-3 trial in South-east Asia, CYD15 = phase-3 trial in Latin America, CYD23 = phase-2b trial in Thailand) for baseline seropositive and baseline seronegative subjects; D) vaccine efficacy estimates by serotype in South-east Asia (i.e. using CYD14 and CYD23 combined) for baseline seropositive and baseline seronegative subjects; E) vaccine efficacy estimates by serotype in Latin America (i.e. using CYD15) for baseline seropositive and baseline seronegative subjects; F) vaccine efficacy estimates by age in South-east Asia (i.e. using CYD14 and CYD23) combined for baseline seropositive and baseline seronegative subjects using 2-5, 6-11 and 12-14 years age-groups; G) vaccine efficacy estimates by age in Latin America (i.e. using CYD15) for baseline seropositive and baseline seronegative subjects using 9-11 and 12-16 years age-groups .

2.5 Association between age and the serotype of infection by pre-exposure

2.5.1 Without imputation

We tested the association between the age-group and the serotype of infection using the Pearson's chi-square test. This analysis was conducted over all trials (i.e. comprising CYD23, CYD14 and CYD15) and on the South East Asian (i.e. CYD23 and CYD14) and Latin American (CYD15) trials separately, for seropositive and seronegative subjects separately and overall.

Supplementary Tables 3-6 provide a descriptive analysis of the number of cases by age-group and by infecting serotype over all trials (using two different age-categorisations), in South East Asia and in Latin America respectively and the results of the Fisher's exact test.

	Overall			Baseline seropositive			Baseline seronegative		
	2-8	9-11	12-16	2-8	9-11	12-16	2-8	9-11	12-16
DENV1	171	147	145	8	11	8	10	6	10
DENV2	166	144	97	10	4	6	10	5	2
DENV3	54	122	71	9	5	7	3	5	1
DENV4	63	90	77	6	7	5	6	4	1
p-value	<0.001			0.720			0.234		

Supplementary Table 3 Overall number of cases occurred during the active surveillance phase of CYD23, CYD14 and CYD15, stratified by age-group (2-8, 9-11, 12-16 years) and infecting serotype. The p-value was calculated using the Fisher's exact test.

	Overall		Baseline seropositive		Baseline seronegative	
	2-8	9-16	2-8	8-16	2-8	9-16
DENV1	171	292	8	19	10	16
DENV2	166	241	10	10	10	7
DENV3	54	193	9	12	3	6
DENV4	63	167	6	12	6	5
p-value	<0.001		0.510		0.486	

Supplementary Table 4 Overall number of cases occurred during the active surveillance phase of CYD23, CYD14 and CYD15, stratified by age-group (2-8, 9-16 years) and infecting serotype. The p-value was calculated using the Fisher's exact test.

	Overall			Baseline seropositive			Baseline seronegative		
	2-5	6-11	12-14	2-5	6-11	12-14	2-5	6-11	12-14
DENV1	96	134	37	4	8	4	5	6	5
DENV2	72	151	20	3	10	4	8	7	1
DENV3	28	49	10	4	6	3	2	3	0
DENV4	31	68	16	3	4	2	5	3	0
p-value	0.115			0.983			0.384		

Supplementary Table 5 Number of cases occurred during the active surveillance phase of CYD23 and CYD14 (South East Asian trials), stratified by age-group (2-5, 6-11, 12-14 years) and infecting serotype. The p-value was calculated using the Fisher's exact test.

	Overall		Baseline seropositive		Baseline seronegative	
	2-11	12-16	2-11	12-16	2-11	12-16
DENV1	88	108	7	4	5	5
DENV2	87	77	1	2	0	1
DENV3	99	61	4	4	3	1
DENV4	54	61	6	3	2	1
p-value	0.009		0.740		0.724	

Supplementary Table 6 Number of cases occurred during the active surveillance phase of CYD15 (Latin American trial), stratified by age-group (2-11, 12-16 years) and infecting serotype. The p-value was calculated using the Fisher's exact.

2.5.2 With imputation

We used the final BRT model to impute the baseline serostatus of the subjects with PD3 PRNT50 titres and missing baseline serostatus and then used the imputed data to test the association between age and the serotype of infection among baseline seropositive and seronegative subjects separately using the Pearson's chi-square test.

We ran 1,000 realizations of the final BRT model and for each realization we obtained a p-value from the Pearson's chi-square test. Supplementary Figure 14 shows the p-value distribution obtained in the 1,000 realisations for each age- and trial- stratification used (the same used in Supplementary section 2.5.1). We used the realizations yielding a p-value <0.05 to further investigate the association between each age-group and serotype. Below we report the significant associations that were obtained in > 50% of the realizations.

Among baseline seropositive subjects in all trials, using 2-8, 9-11, 12-16 years age-groups we found that 92.4% of realizations yielded a significant (p-value < 0.05) association, with more DENV2 and fewer DENV3 infections among 2-8 year olds than expected in 85.7% and 79.0% of realizations respectively. Using 2-8 and 9-16 years age-groups, we found a significant (p-value < 0.05) association in 94.6% of realizations, with more DENV2 and fewer DENV3 infections among 2-8 year olds than expected in 85.7% and 79.0% of realizations respectively.

Among baseline seronegative subjects in all trials, using 2-8, 9-11, 12-16 years age-groups we found that 84.5% of realizations yielded a significant (p-value < 0.05) association, with more DENV3 infections among 9-11 year olds than expected in 73.2% of realizations. Using 2-8 and 9-16 years age-groups, we found a significant (p-value < 0.05) association in 64.9% of realizations. However, significant associations among each age-group and serotype were detected in < 50% of the realizations.

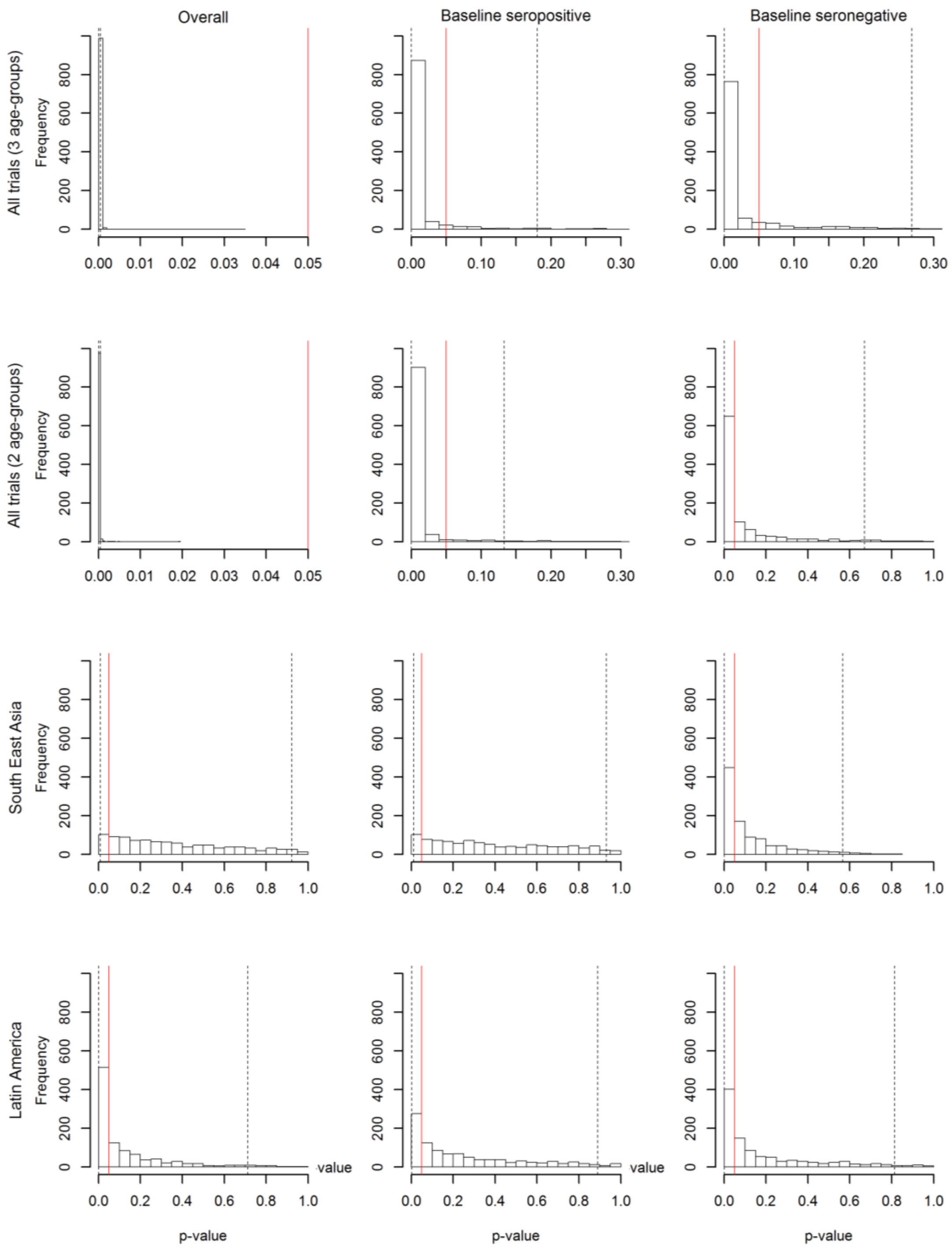

Supplementary Figure 14 P-value distributions obtained with the Pearson's chi-squared test and imputation of the baseline serostatus from 1,000 realizations of the final BRT model trained on 50% of non-cases and 75% of cases and using 10-fold cross-validation, tc = 16, lr = 0.0005, bf = 0.75. The vertical dashed lines represent the 2.5 and 97.5 percentiles. The vertical red line denotes the significance level value of 0.05.

2.6 Age-trends in vaccine efficacy

We tested the significance of age-trends in the vaccine estimates obtained in 3 age-groups (both without imputation and with imputation of the baseline serostatus) using weighted linear regression. In this analysis, we used the average estimated efficacy for each of three age-groups as the response variable, the mid-point of the age-groups as the predictor and the reciprocal of the efficacies' variance as the weight.

The significance of the age-trends in the vaccine estimates obtained using 2 age-groups (both without imputation and with imputation of the baseline serostatus) was tested using the Pearson's chi-square test with significance level 0.05.

2.6.1 Without imputation

Supplementary Table 7 shows the estimated coefficients from weighted linear regression obtained without imputation and using a significance level of 0.05, for baseline seropositive and baseline seronegative subjects separately, using all trials (with age-groups 2-8, 9-11 and 12-16 years) and the South East Asian trials (CYD23 and CYD14) (with age-groups 2-5, 6-11 and 12-14 years). Supplementary Figure 15 shows the estimated age-trend in vaccine efficacy among baseline seronegative subjects over all trials.

We tested the statistical significance of the difference between the vaccine efficacy estimated among subjects 2-8 and 9-16 years old over all trials and among subjects 2-11 and 12-16 years old in Latin America using a chi-squared test, where the variance of the vaccine efficacy estimates was calculated by bootstrapping (Supplementary Table 8).

	Baseline seropositive				Baseline seronegative			
	All trials		South East Asia		All trials		South East Asia	
	mean	95% CI	mean	95% CI	mean	95% CI	mean	95%CI
Slope	0.01	(-0.06, 0.08)	0.020	(-0.13, 0.17)	**0.046**	**(0.004, 0.09)**	0.08	(-0.31, 0.47)
Intercept	0.66	(-0.12, 1.43)	0.56	(-1.03, 2.16)	-0.11	(-0.59, 0.37)	-0.25	(-4.95, 4.45)

Supplementary Table 7 Estimated coefficients of weighted linear regression with average efficacy as response variable, age-group mid-points as predictors and the reciprocal of efficacies' variance as weights. Significance level 0.05, F-test.

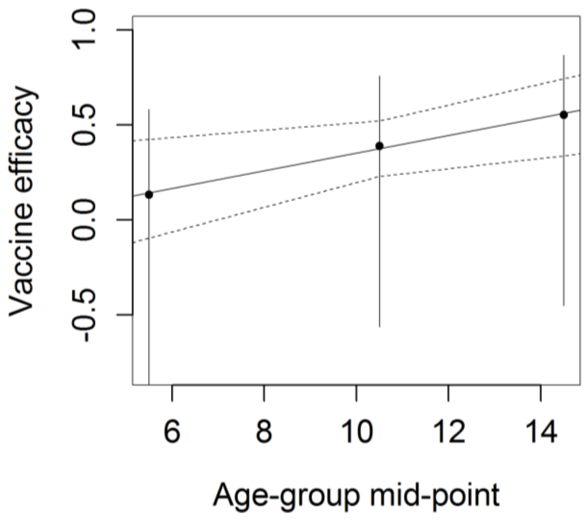

Supplementary Figure 15 Mean (solid line) and 95% confidence interval (dashed lines) of the age-trend in the estimated vaccine efficacy among baseline seronegative subjects in all trials, using age-groups 2-8, 9-11 and 12-16 years, without imputation.

	Baseline seropositive				Baseline seronegative			
	All trials		Latin America		All trials		Latin America	
	VE 2-8	VE 9-16	VE 2-11	VE 12-16	VE 2-8	VE 9-16	VE 2-11	VE 12-16
Mean	0.71	0.82	0.87	0.79	0.13	0.47	0.58	0.18
Variance	1.14	0.33	0.75	3.25	20.35	4.04	19.61	71.41
p-value	0.373		0.683		0.498		0.677	

Supplementary Table 8 Vaccine efficacy estimates without imputation among 2-8 and 9-16 year olds in all trials (CYD23, CYD14 and CYD15) and among 2-11 and 12-16 year olds in Latin America (CYD15). The variance has been calculated by bootstrapping. We assessed the statistical difference in the estimated vaccine efficacies between the age-groups (p-value) using the chi-squared test.

2.6.2 With imputation

Supplementary Table 9 shows the estimated coefficients from weighted linear regression obtained with imputation of the baseline serostatus using the final BRT model. We used a significance level of 0.05 and performed the analysis for baseline seropositive and baseline seronegative subjects separately, using all trials (with age-groups 2-8, 9-11 and 12-16 years) and the South East Asian trials (CYD23 and CYD14) (with age-groups 2-5, 6-11 and 12-14 years). Supplementary Figure 16 shows the estimated age-trend in vaccine efficacy among baseline seronegative subjects in the South East Asian trials.

We tested the statistical significance of the difference between the vaccine efficacy estimates obtained with imputation among subjects 2-8 and 9-16 years old over all trials and among subjects 2-11 and 12-16 years old in Latin America using the chi-squared test (Supplementary Table 10), where the mean and variance of the imputed vaccine efficacy estimates in each age-group were calculated from 1,000 realisations of the final BRT model. None of the chi-square statistics calculated in this analysis were significant using significance level 0.05 (Supplementary Table 10).

In a sensitivity analysis we tested the significance of the age-trend in vaccine efficacy obtained using all trials and a finer age-stratification into 2-years age-groups (i.e. 2-3, 4-5, 6-7, 8-9, 10-11, 12-13 and 14-16 years). Supplementary Table 11 shows the estimated coefficients from weighted linear regression obtained with imputation using 2-years age-groups and Supplementary Figure 17 shows the fit of the estimated linear trend.

	Baseline seropositive				Baseline seronegative			
	All trials		South East Asia		All trials		South East Asia	
	mean	95% CI	mean	95% CI	mean	95% CI	mean	95%CI
Slope	0.02	(-0.04, 0.09)	0.03	(-0.05, 0.11)	0.03	(-0.03, 0.09)	**0.047**	**(0.0005, 0.09)**
Intercept	0.43	(-0.39, 1.26)	0.37	(-0.51 1.26)	0.09	(-0.60, 0.78)	-0.04	(-0.48, 0.39)

Supplementary Table 9 Estimated coefficients of weighted linear regression with average efficacy as response variable, age-group mid-points as predictors and the reciprocal of efficacies' variance as weights. Significance level 0.05, F-test.

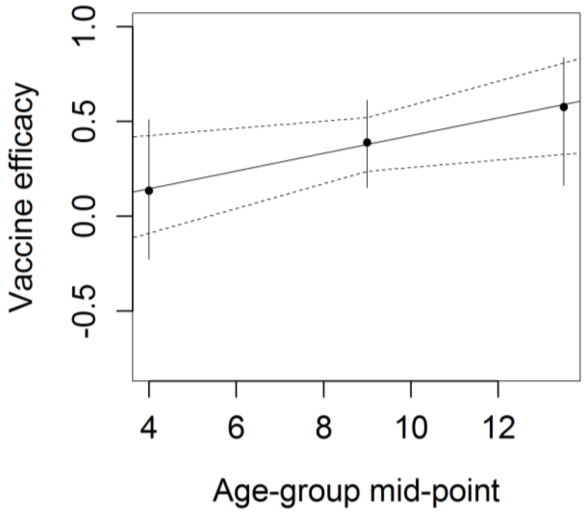

Supplementary Figure 16 Mean (solid line) and 95% confidence interval (dashed lines) of the age-trend in the estimated vaccine efficacy among baseline seronegative subjects in the South East Asian trials (CYD23 and CYD14), using age-groups 2-5, 6-11 and 12-14 years, with imputation of the baseline serostatus from 1,000 realizations of the final BRT model trained on 50% of non-cases and 75% of cases and using 10-fold cross-validation, tc = 16, lr = 0.0005, bf = 0.75.

	Baseline seropositive				Baseline seronegative			
	All trials		Latin America		All trials		Latin America	
	VE 2-8	VE 9-16	VE 2-11	VE 12-16	VE 2-8	VE 9-16	VE 2-11	VE 12-16
Mean	0.544	0.726	0.710	0.758	0.262	0.444	0.382	0.470
Variance	0.013	0.002	0.003	0.003	0.018	0.006	0.013	0.009
P-value	0.128		0.531		0.234		0.544	

Supplementary Table 10 Vaccine efficacy estimates with imputation among 2-8 and 9-16 year olds in all trials (CYD23, CYD14 and CYD15) and among 2-11 and 12-16 year olds in Latin America (CYD15). We assessed the statistical difference in the estimated vaccine efficacies between the age-groups using the chi-squared test.

	Baseline seropositive		Baseline seronegative	
	mean	95% CI	mean	95% CI
Slope	0.013	(-0.019, 0.045)	**0.029**	**(0.004, 0.053)**
Intercept	0.578	(0.203, 0.955)	0.107	(-0.189, 0.403)

Supplementary Table 11 Estimated coefficients of weighted linear regression with average efficacy as response variable, age-group mid-points as predictors and the reciprocal of efficacies' variance as weights. Significance level 0.05, F-test.

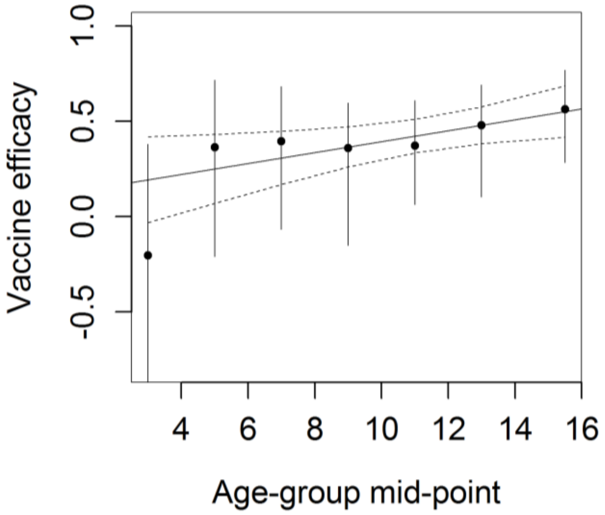

Supplementary Figure 17 Mean (solid line) and 95% confidence interval (dashed lines) of the age-trend in the estimated vaccine efficacy among baseline seronegative subjects in all trials (CYD23, CYD14 and CYD15), using age-groups 2-3, 4-5, 6-

7, 8-9, 10-11, 12-13 and 14-16 years, with imputation of the baseline serostatus from 1,000 realizations of the final BRT model trained on 50% of non-cases and 75% of cases and using 10-fold cross-validation, tc = 16, lr = 0.0005, bf = 0.75.

2.7 Percentage increase in variance due to incomplete baseline serostatus among subjects with PD3 titres

We present in Supplementary Table 12 the estimates of the relative increase in variance due to incomplete baseline serostatus among the subjects with observed PD3 titres. Relative increases in variance, given by $(F - \bar{C}) / \bar{C}$, are provided for the main vaccine efficacy estimates presented in Figure 1 and were calculated using the method described in Supplementary section 1.2.6.

**Vaccine Efficacy (VE)**	**F**	$\bar{C}$	$(F - \bar{C}) / \bar{C}$
VE in baseline seropositive subjects (Fig. 1B)	0.0027	0.0007	308.15%
VE in baseline seronegative subjects (Fig. 1B)	0.0067	0.0030	126.41%
VE in baseline seropositive 2-8 year olds (Fig. 1C)	0.0143	0.0053	171.60%
VE in baseline seronegative 2-8 year olds (Fig. 1C)	0.0215	0.0113	89.73%
VE in baseline seropositive 9-11 year olds (Fig. 1C)	0.0028	0.0015	89.53%
VE in baseline seronegative 9-11 year olds (Fig. 1C)	0.0121	0.0089	35.65%
VE in baseline seropositive 12-16 year olds (Fig. 1C)	0.0022	0.0013	70.85%
VE in baseline seronegative 12-16 year olds (Fig. 1C)	0.0097	0.0071	37.16%
VE in baseline seropositive 9-16 year olds (Fig. 1C)	0.0017	0.0007	148.53%
VE in baseline seronegative 9-16 year olds (Fig. 1C)	0.0069	0.0039	74.97%
VE against DENV1 in baseline seropositive 2-16 year olds (Fig. 1D)	0.0082	0.0049	68.08%
VE against DENV1 in baseline seronegative 2-16 year olds (Fig. 1D)	0.0108	0.0047	130.03%
VE against DENV2 in baseline seropositive 2-16 year olds (Fig. 1D)	0.0032	0.0022	50.27%
VE against DENV2 in baseline seronegative 2-16 year olds (Fig. 1D)	0.0015	0.0009	60.64%
VE against DENV3 in baseline seropositive 2-16 year olds (Fig. 1D)	0.0107	0.0066	62.50%
VE against DENV3 in baseline seronegative 2-16 year olds (Fig. 1D)	0.0720	0.0562	28.05%
VE against DENV4 in baseline seropositive 2-16 year olds (Fig. 1D)	0.0161	0.0095	69.15%
VE against DENV4 in baseline seronegative 2-16 year olds (Fig. 1D)	0.0256	0.0220	16.38%
VE against DENV1 in baseline seropositive 2-8 year olds (Fig. 1E)	0.0655	0.0487	34.46%
VE against DENV1 in baseline seronegative 2-8 year olds (Fig. 1E)	0.0473	0.0325	45.47%
VE against DENV2 in baseline seropositive 2-8 year olds (Fig. 1E)	0.0406	0.0349	16.23%
VE against DENV2 in baseline seronegative 2-8 year olds (Fig. 1E)	0.0311	0.0203	53.17%
VE against DENV3 in baseline seropositive 2-8 year olds (Fig. 1E)	0.0354	0.0275	28.70%
VE against DENV3 in baseline seronegative 2-8 year olds (Fig. 1E)	0.1467	0.1337	9.78%
VE against DENV4 in baseline seropositive 2-8 year olds (Fig. 1E)	0.0715	0.0586	21.97%
VE against DENV4 in baseline seronegative 2-8 year olds (Fig. 1E)	0.3557	0.2991	18.94%
VE against DENV1 in baseline seropositive 9-16 year olds (Fig. 1F)	0.0081	0.0058	38.68%
VE against DENV1 in baseline seronegative 9-16 year olds (Fig. 1F)	0.0080	0.0051	59.01%
VE against DENV2 in baseline seropositive 9-16 year olds (Fig. 1F)	0.0034	0.0024	45.19%
VE against DENV2 in baseline seronegative 9-16 year olds (Fig. 1F)	0.0011	0.0008	44.86%
VE against DENV3 in baseline seropositive 9-16 year olds (Fig. 1F)	0.0128	0.0091	41.30%
VE against DENV3 in baseline seronegative 9-16 year olds (Fig. 1F)	0.2159	0.1751	23.28%
VE against DENV4 in baseline seropositive 9-16 year olds (Fig. 1F)	0.0228	0.0157	44.71%
VE against DENV4 in baseline seronegative 9-16 year olds (Fig. 1F)	0.0296	0.0281	5.41%

Supplementary Table 12 Estimates of the vaccine efficacies' variance (F and $\bar{C}$, as described in Supplementary section 1.2.6), percentage increase in variance due to missing baseline serostatus among the subjects with given PD3 titres ($(F - \bar{C}) / \bar{C}$,) from 1,000 realisations of the final BRT model trained on 50% of non-cases and 75% of cases and using 10-fold cross-validation, tc = 16, lr = 0.0005, bf = 0.75.

2.8 Sensitivity Analysis

2.8..1 Vaccine efficacy estimates obtained including individual-level imputations only

We tested the effect that the group-level inference of the baseline serostatus had on the vaccine efficacy estimates. In Supplementary Figures 18 and 19 we show the vaccine efficacy estimates by baseline serostatus obtained on 4,918 subjects, i.e. the 4,251 subjects with observed baseline status and the 667 subjects with imputed baseline serostatus only, without including the group-level inference of the baseline serostatus for the 30,769 subjects with missing PD3 PRNT50 titres. The estimates in Supplementary Figures 18 and 19 were obtained from 1,000 realisations of the final BRT model and the optimal hyper-parameterisation (tc = 16, lr = 0.0005 and bf = 0.75), with training sets generated by sampling 50% of the non-cases and 75% of the cases.

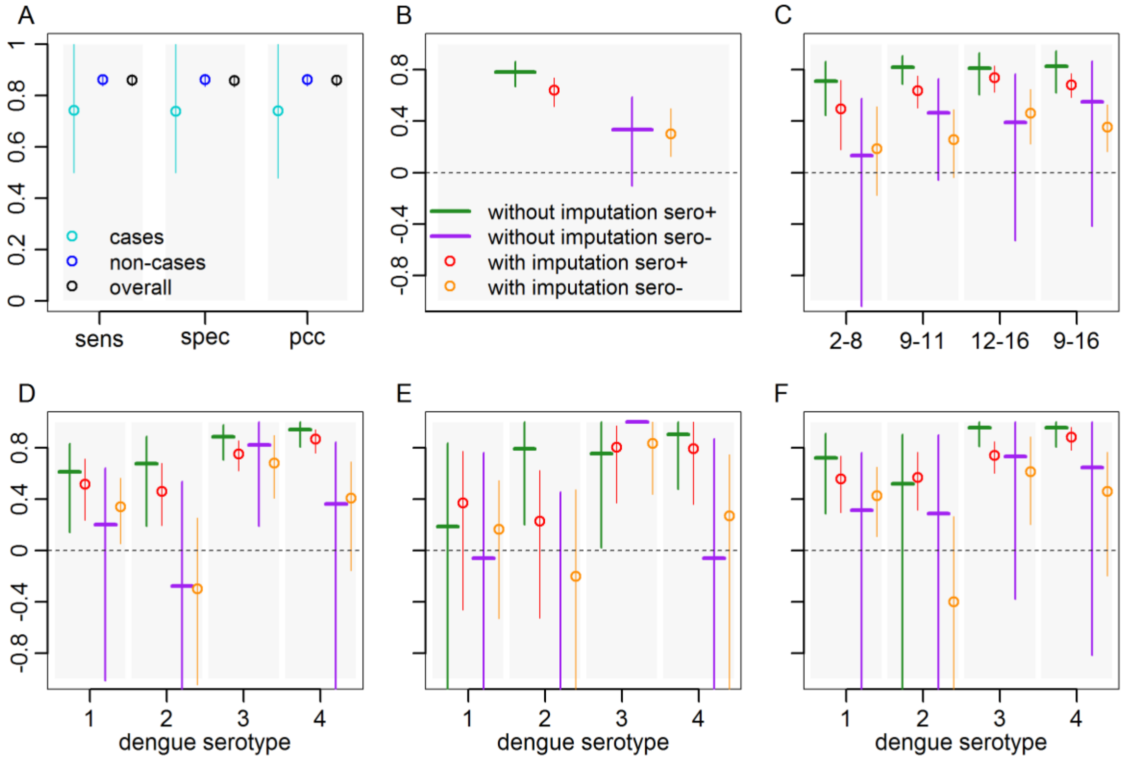

Supplementary Figure 18 Mean and 95% CI of the vaccine efficacy estimates generated with and without imputed data for baseline seronegative (sero-) and baseline seropositive (sero+) subjects separately. Estimates were obtained from 1,000 realisations of the final BRT model trained on 50% of non-cases and 75% of cases and using 10-fold cross-validation, tc = 16, lr = 0.0005, bf = 0.75, having included individual-level imputations of the baseline serostatus for 667 subjects only (i.e. without group-level inference of the baseline serostatus). A) sensitivity (sens), specificity (spec) and proportion of correct classifications (pcc) among cases, non-cases and overall; B) vaccine efficacy estimates for baseline seropositive and baseline seronegative subjects; C) vaccine efficacy estimates for baseline seropositive and baseline seronegative subjects by age using 2-8, 9-11, 12-16 and 9-16 years age-groups; D) vaccine efficacy estimates by serotype for baseline seropositive and baseline seronegative subjects of all ages (2-16 years); E) vaccine efficacy estimates by serotype for baseline seropositive and baseline seronegative subjects 2-8 years old; F) vaccine efficacy estimates by serotype for baseline seropositive and baseline seronegative subjects 9-16 years old.

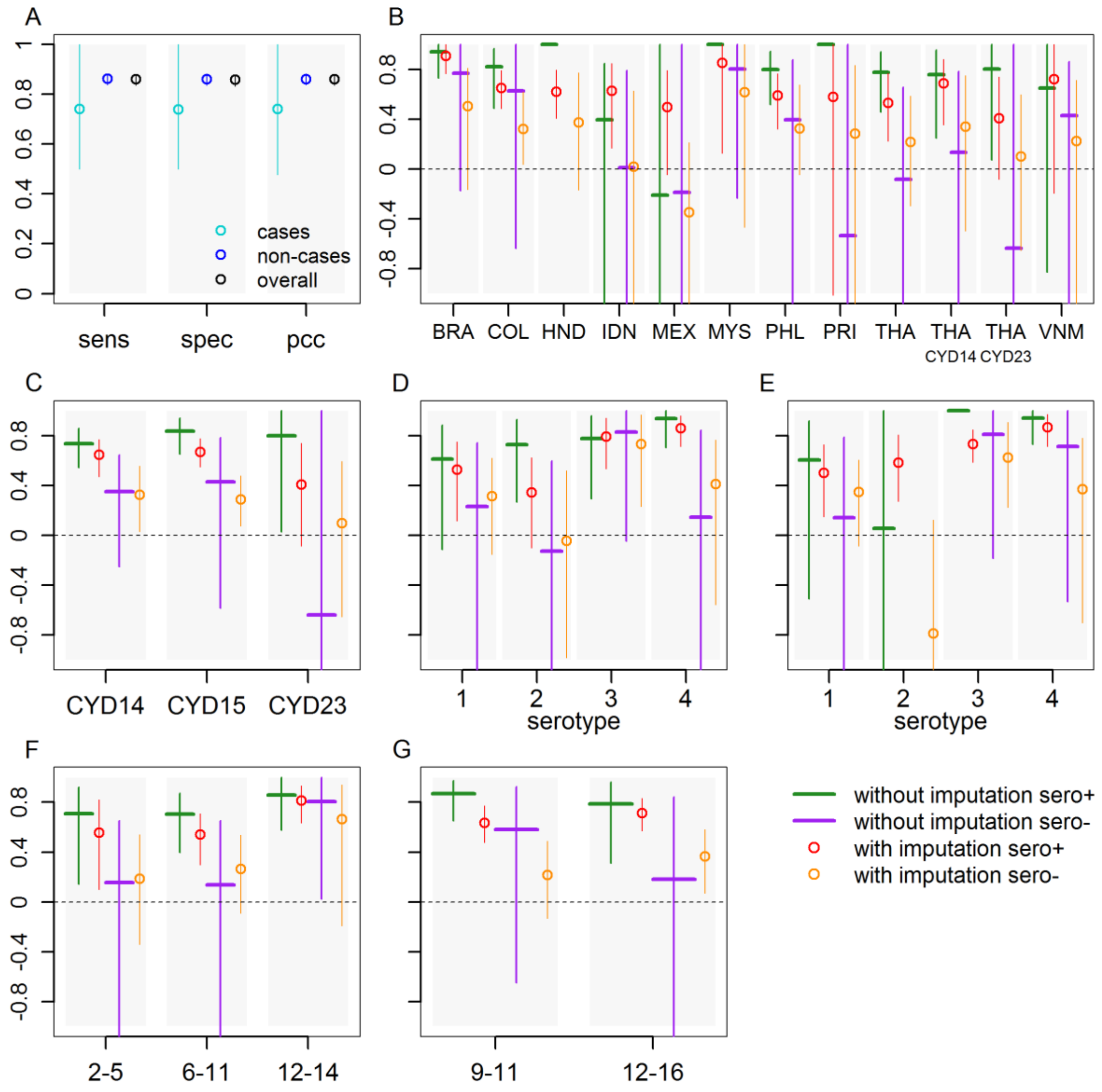

Supplementary Figure 19 Mean and 95% CI of vaccine efficacy estimates generated with and without imputed data for baseline seronegative (sero-) and baseline seropositive (sero+) subjects. Estimates were obtained from 1,000 realisations of the final BRT model trained on 50% of non-cases and 75% of cases and using 10-fold cross-validation, tc = 16, lr = 0.0005, bf = 0.75, having included individual-level imputations of the baseline serostatus for 667 subjects only (i.e. without group-level inference of the baseline serostatus included). A) sensitivity (sens), specificity (spec) and proportion of correct classifications (pcc) among cases, non-cases and overall; B) vaccine efficacy estimates by country for baseline seropositive and baseline seronegative subjects (BRA = Brazil, OL = Colombia, HND = Honduras, IDN = Indonesia, MEX = Mexico, MYS = Malaysia, PHL = Philippines, PRI = Puerto Rico, THA = Thailand, VNM = Vietnam). Efficacy estimates for Thailand were generated using the phse-2b (CYD23) and the phase-3 (CYD14) data separately and combined; C) vaccine efficacy estimates by trial (CYD14 = phase-3 trial in South-east Asia, CYD15 = phase-3 trial in Latin America, CYD23 = phase-2b trial in Thailand) for baseline seropositive and baseline seronegative subjects; D) vaccine efficacy estimates by serotype in South-east Asia (i.e. using CYD14 and CYD23 combined) for baseline seropositive and baseline seronegative subjects; E) vaccine efficacy estimates by serotype in Latin America (i.e. using CYD15) for baseline seropositive and baseline seronegative subjects; F) vaccine efficacy estimates by age in South-east Asia (i.e. using CYD14 and CYD23) combined for baseline seropositive and baseline seronegative subjects using 2-5, 6-11 and 12-14 years age-groups; G) vaccine efficacy estimates by age in Latin America (i.e. using CYD15) for baseline seropositive and baseline seronegative subjects using 9-11 and 12-16 years age-groups .

2.8.2 Vaccine efficacy estimates calculated on the phase-3 trials only

Supplementary Figure 20 shows the vaccine efficacy estimates calculated on phase-3 trials (CYD14 and CYD15) data, having trained the BRT model on all available data, including the phase-2b trial (CYD23) data.

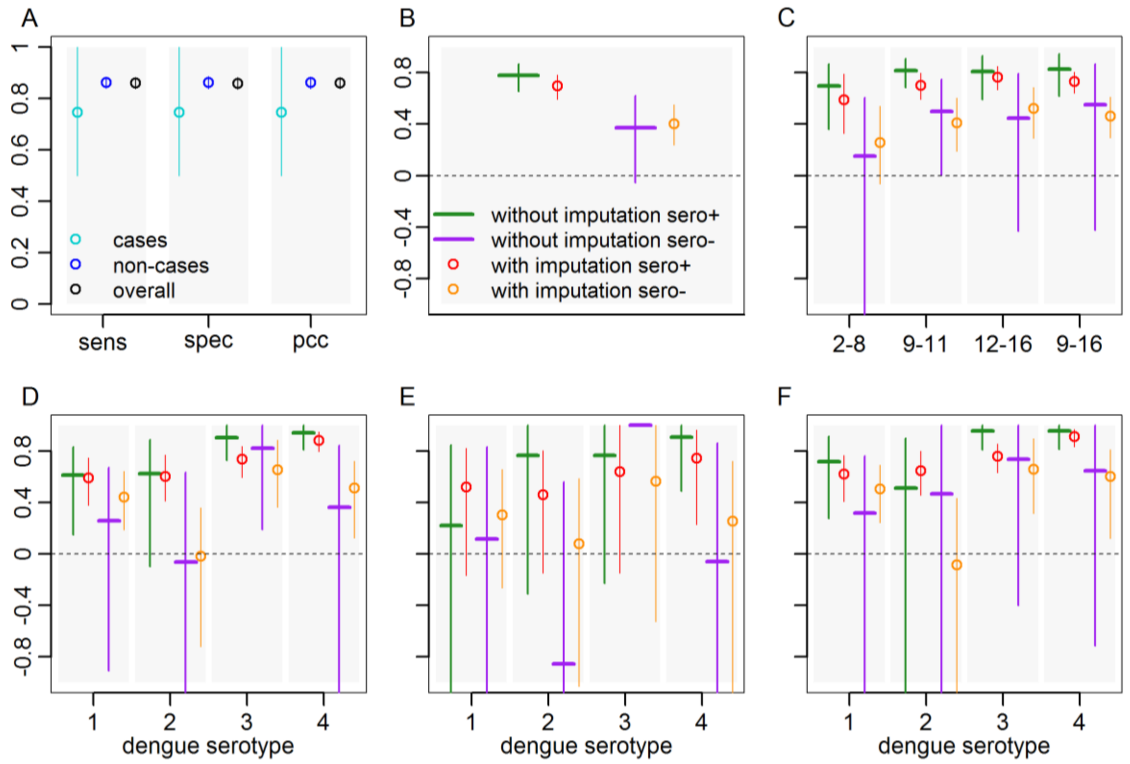

Supplementary Figure 20 Mean and 95% CI of the vaccine efficacy estimates generated with and without imputed data for baseline seronegative (sero-) and baseline seropositive (sero+) subjects enrolled in the phase-3 trails (CYD14 & CYD15), having fitted the BRT model on all available data including the phase-2b trial (CYD23). Estimates were obtained from 1,000 realisations of the final BRT model, using 10-fold cross-validation, tc = 16, lr = 0.0005, bf = 0.75 and by randomly sampling 50% of non-cases and 75% of cases to build the training set. A) sensitivity (sens), specificity (spec) and proportion of correct classifications (pcc) among cases, non-cases and overall; B) vaccine efficacy estimates for baseline seropositive and baseline seronegative subjects; C) vaccine efficacy estimates for baseline seropositive and baseline seronegative subjects by age using 2-8, 9-11, 12-16 and 9-16 years age-groups; D) vaccine efficacy estimates by serotype for baseline seropositive and baseline seronegative subjects of all ages (2-16 years); E) vaccine efficacy estimates by serotype for baseline seropositive and baseline seronegative subjects 2-8 years old; F) vaccine efficacy estimates by serotype for baseline seropositive and baseline seronegative subjects 9-16 years old.

2.8.3 Vaccine efficacy estimates based on the phase-3 trials only

Supplementary Figure 21 shows the vaccine efficacy estimates based on the phase-3 trials (CYD14 and CYD15) data only, including BRT model training (i.e. no use of the phase-2b trial (CYD23) data was made).

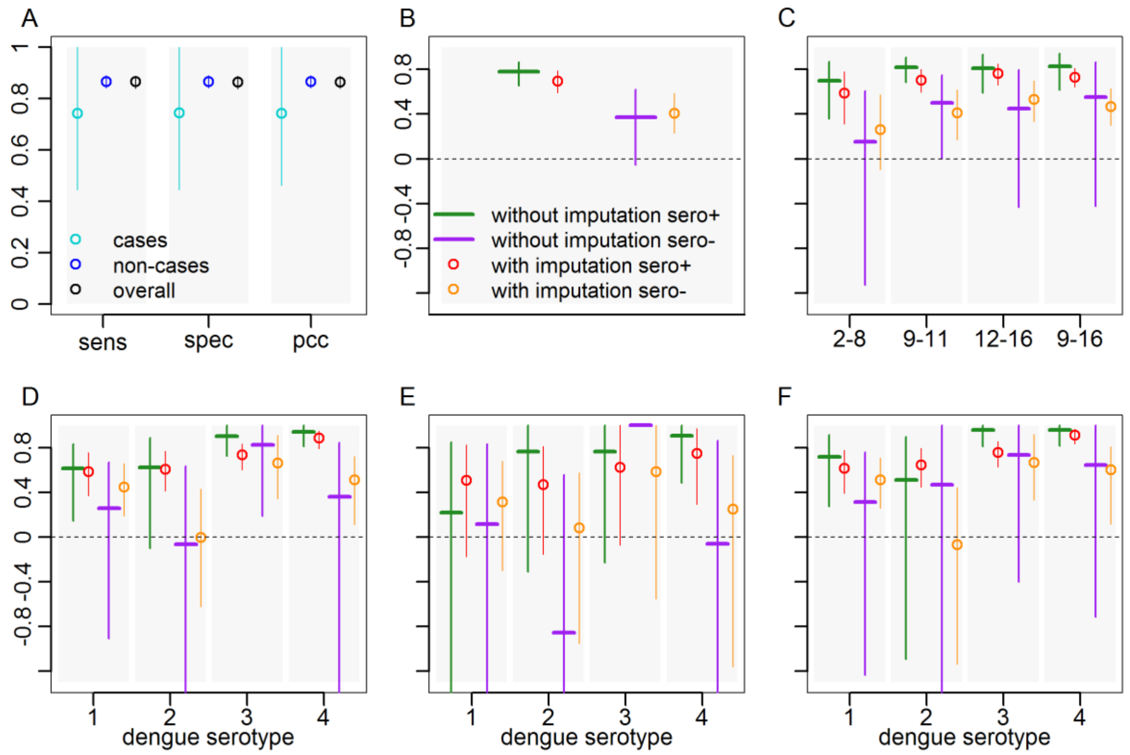

Supplementary Figure 21 Mean and 95% CI of the vaccine efficacy estimates generated with and without imputed data for baseline seronegative (sero-) and baseline seropositive (sero+) subjects enrolled in the phase-3 trails (CYD14 & CYD15) (no use of the phase-2b data was made). Estimates were obtained from 1,000 realisations of the final BRT model, using 10-fold cross-validation, tc = 16, lr = 0.0005, bf = 0.75 and by randomly sampling 50% of non-cases and 75% of cases to build the training set.A) sensitivity (sens), specificity (spec) and proportion of correct classifications (pcc) among cases, non-cases and overall; B) vaccine efficacy estimates for baseline seropositive and baseline seronegative subjects; C) vaccine efficacy estimates for baseline seropositive and baseline seronegative subjects by age using 2-8, 9-11, 12-16 and 9-16 years age-groups; D) vaccine efficacy estimates by serotype for baseline seropositive and baseline seronegative subjects of all ages (2-16 years); E) vaccine efficacy estimates by serotype for baseline seropositive and baseline seronegative subjects 2-8 years old; F) vaccine efficacy estimates by serotype for baseline seropositive and baseline seronegative subjects 9-16 years old.

2.8.4 Vaccine efficacy estimates for seronegative, monotypic and multitypic PRNT50 profiles

We tested the effect of refining the definition of pre-exposure by further dividing the subjects classified as seropositive into monotypic or multitypic. Specifically, we classified the subjects with only one PRNT50 titre > 10 or, if more than one PRNT50 titre > 10 were present, only a single PRNT50 titre > 80 as monotypic (i.e. likely exposed to a single dengue infection) and the subjects with more than one PRNT50 titre > 10 and more than one PRNT50 titre > 80 as multitypic (i.e. likely exposed to two or more dengue infections). The subjects with all four PRNT50 titres < 10 were classified as baseline seronegative, as in the previous analysis.

Using these definitions, we searched for the optimal hyper-parameterisation of BRT models using the scenarios described in Supplementary Table 1. For each scenario (Supplementary Table 1), we tested 54 different hyper-parameterisations that derived from the combination of 9 tree-complexities (*tc* = 1, 5, 10, 15, 20, 25, 30, 35, 40), 3 learning rates (*lr* = 0.01, 0.005, 0.001) and 2 bag fractions (*bf* = 0.5, 0.75). For each of the 486 (= 9 x 54) models defined, we ran 100 random realisations using the algorithm described in Supplementary section 1.2.2 (in step 3 we used the gbm function in the gbm[8] package in place of the gbm.step function in the dismo[6] package, as this latter does not include the multinomial family) and computed the out-of-sample proportion of seronegative, monotypic and multitypic PRNT50 profiles correctly classified in step 7. The BRT models were fitted using the PD3 PRNT50 titres, country, age, gender, the infecting serotype and the time interval between dose 3 and symptoms onset as predictors, i.e. the optimal set of predictors retrieved from our previous analysis.

Supplementary Figures 22-30 show the out-of-sample proportions of seronegative, monotypic and multitypic profiles correctly classified among cases, non-cases and overall. Following Elith et al.[4], we only retained models with at least 1,000 trees. Visual inspection of Supplementary Figures 22-30 suggested that the model with tree complexity (*tc*) = 15, learning rate (*lr*) = 0.001, bag fraction (*bf*) = 0.75 and training sets generated by sampling 75% of the cases and 75% of the non-cases achieved among the highest out-of-sample proportion of correct classification among seronegative, monotypic and multitypic cases. The out-of-sample proportion of seronegative and multitypic profiles correctly classified among non-cases was relatively high compared to the relative proportions among cases. Using this optimal parameterisation we estimated vaccine efficacy using the formulas and algorithm described in Supplementary sections 1.2.4 and 1.2.5, adapted to the multinomial cases. Supplementary Figures 31-33 show the observed and estimated proportions of seronegative, monotypic and multitypic PRNT50 profiles among cases and non-cases by country and age-group. The corresponding vaccine efficacy estimates are shown in Figure 2 of the main text and in Supplementary Figure 34. From 1,000 random realisations of the BRT model trained on training sets generated by randomly sampling 75% of the non-cases and 75% of the cases and using *tc* = 15, *lr* = 0.001 and *bf* = 0.75, we estimated the mean relative contributions presented in Supplementary Table 13.

Predictors	BRT model
PD3 PRNT50 titre against DENV1	38.9
PD3 PRNT50 titre against DENV2	15.4
PD3 PRNT50 titre against DENV3	12.8
Country	9.3
Age	8.9
PD3 PRNT50 titre against DENV4	7.6
Arm	4.8
Serotype	1.0
Time interval between dose 3 to symptoms	0.8
Gender	0.5

Supplementary Table 13 Relative contributions (%) of the predictors used in BRT models using 10-fold cross-validation, tc = 15, lr = 0.001 and bf = 0.75, and trained on 100 training sets generated by randomly sampling 75% of non-cases and 75% of cases with observed baseline serostatus.

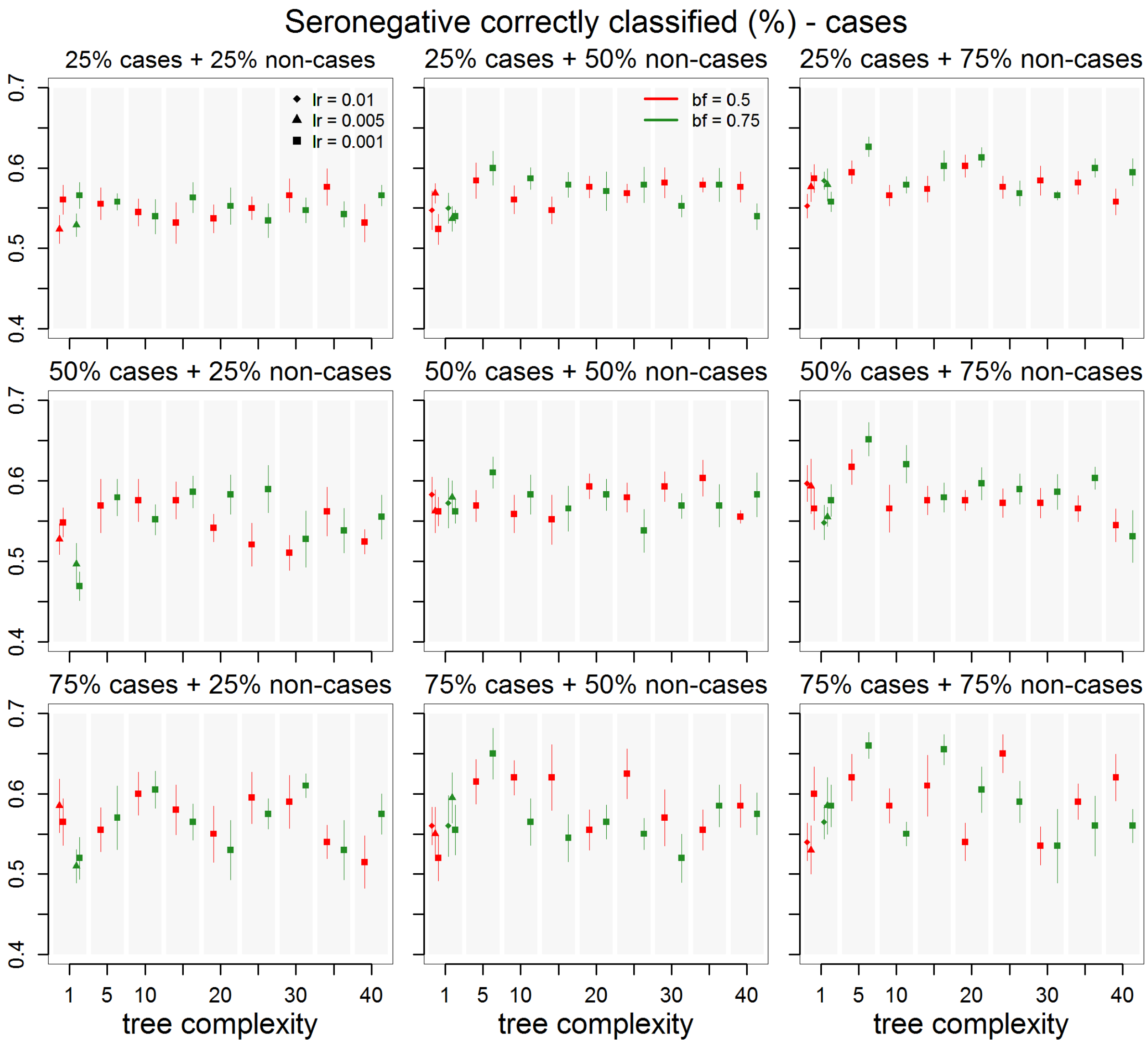

Supplementary Figure 22 Out-of-sample proportion of seronegative cases correctly classified (mean and 95% CI) using all combinations of tree complexities (tc = 1, 5, 10, 15, 20, 25, 30, 35, 40), learning rates (lr = 0.01, 0.005, 0.001), bag fractions (bf = 0.5, 0.75) and training sets (see Supplementary Table 1) from 10 randomly sampled training and validation sets (Supplementary section 1.2.2). Only BRT models with at least 1,000 trees are shown.

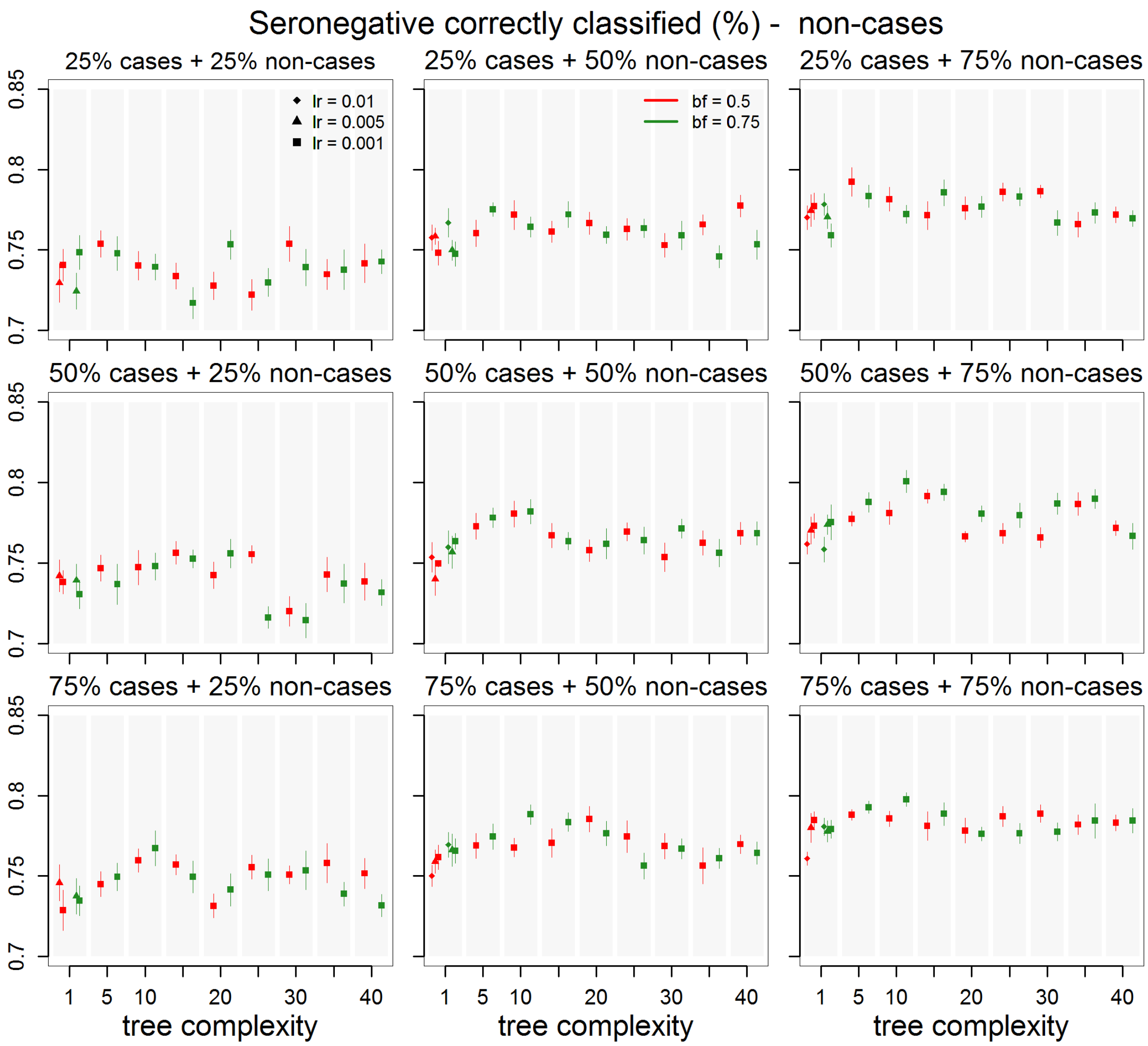

Supplementary Figure 23 Out-of-sample proportion of seronegative non-cases correctly classified (mean and 95% CI) using all combinations of tree complexities (tc = 1, 5, 10, 15, 20, 25, 30, 35, 40), learning rates (lr = 0.01, 0.005, 0.001), bag fractions (bf = 0.5, 0.75) and training sets (see Supplementary Table 1) from 10 randomly sampled training and validation sets (Supplementary section 1.2.2). Only BRT models with at least 1,000 trees are shown.

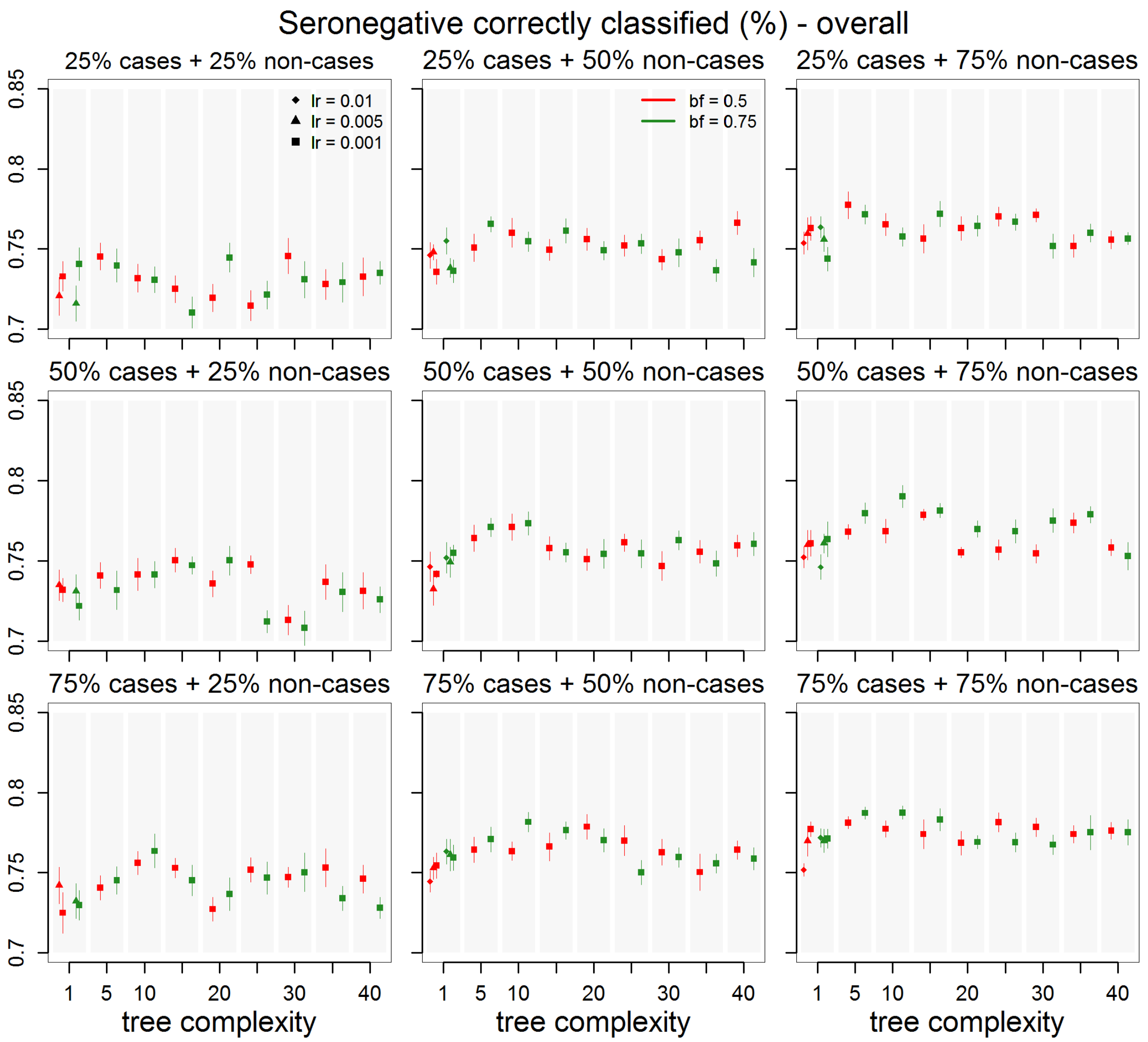

Supplementary Figure 24 Out-of-sample proportion of seronegative cases and non-cases (overall) correctly classified (mean and 95% CI) using all combinations of tree complexities (tc = 1, 5, 10, 15, 20, 25, 30, 35, 40), learning rates (lr = 0.01, 0.005, 0.001), bag fractions (bf = 0.5, 0.75) and training sets (see Supplementary Table 1) from 10 randomly sampled training and validation sets (Supplementary section 1.2.2). Only BRT models with at least 1,000 trees are shown.

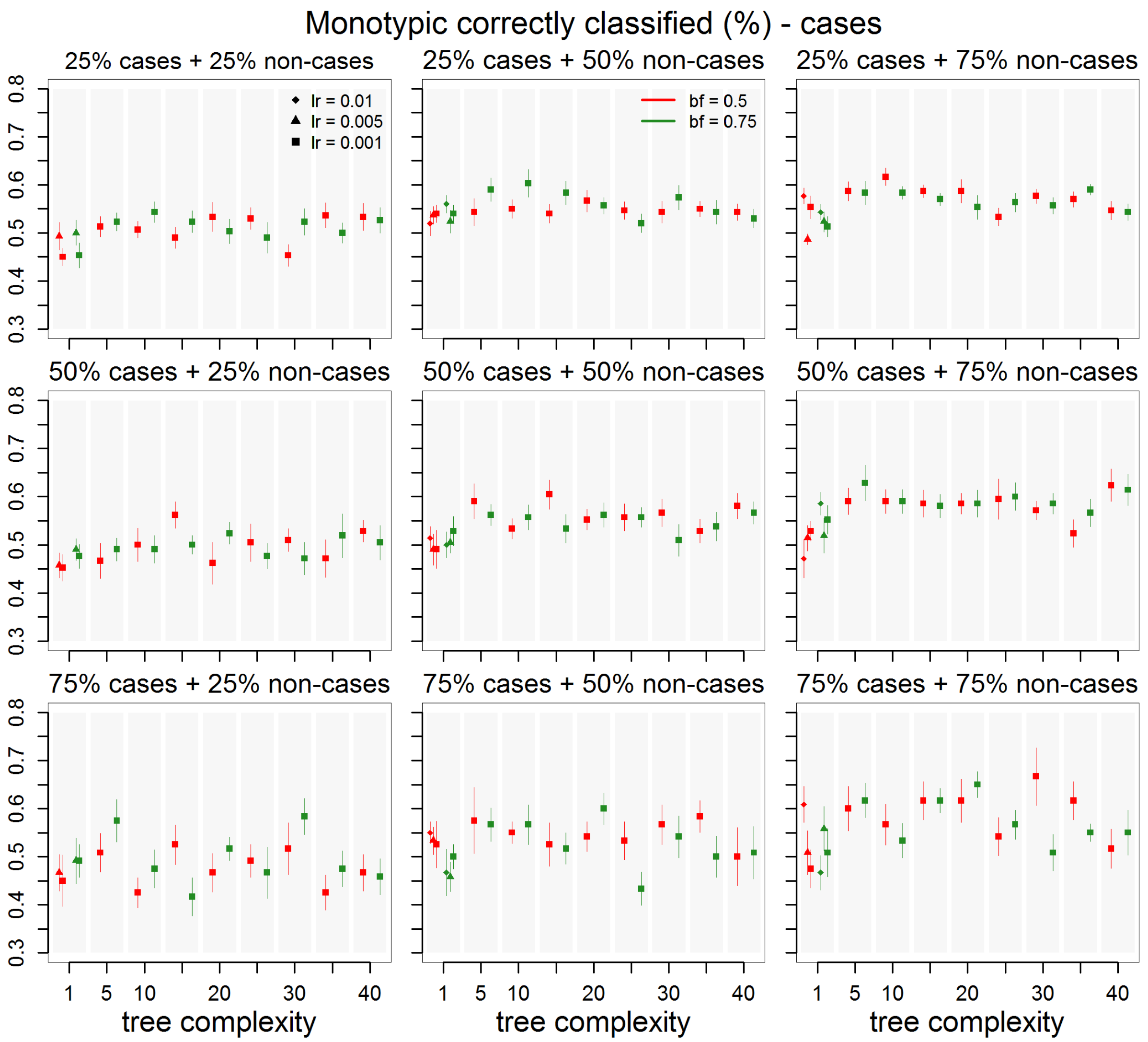

Supplementary Figure 25 Out-of-sample proportion of monotypic cases correctly classified (mean and 95% CI) using all combinations of tree complexities (tc = 1, 5, 10, 15, 20, 25, 30, 35, 40), learning rates (lr = 0.01, 0.005, 0.001), bag fractions (bf = 0.5, 0.75) and training sets (see Supplementary Table 1) from 10 randomly sampled training and validation sets (Supplementary section 1.2.2). Only BRT models with at least 1,000 trees are shown.

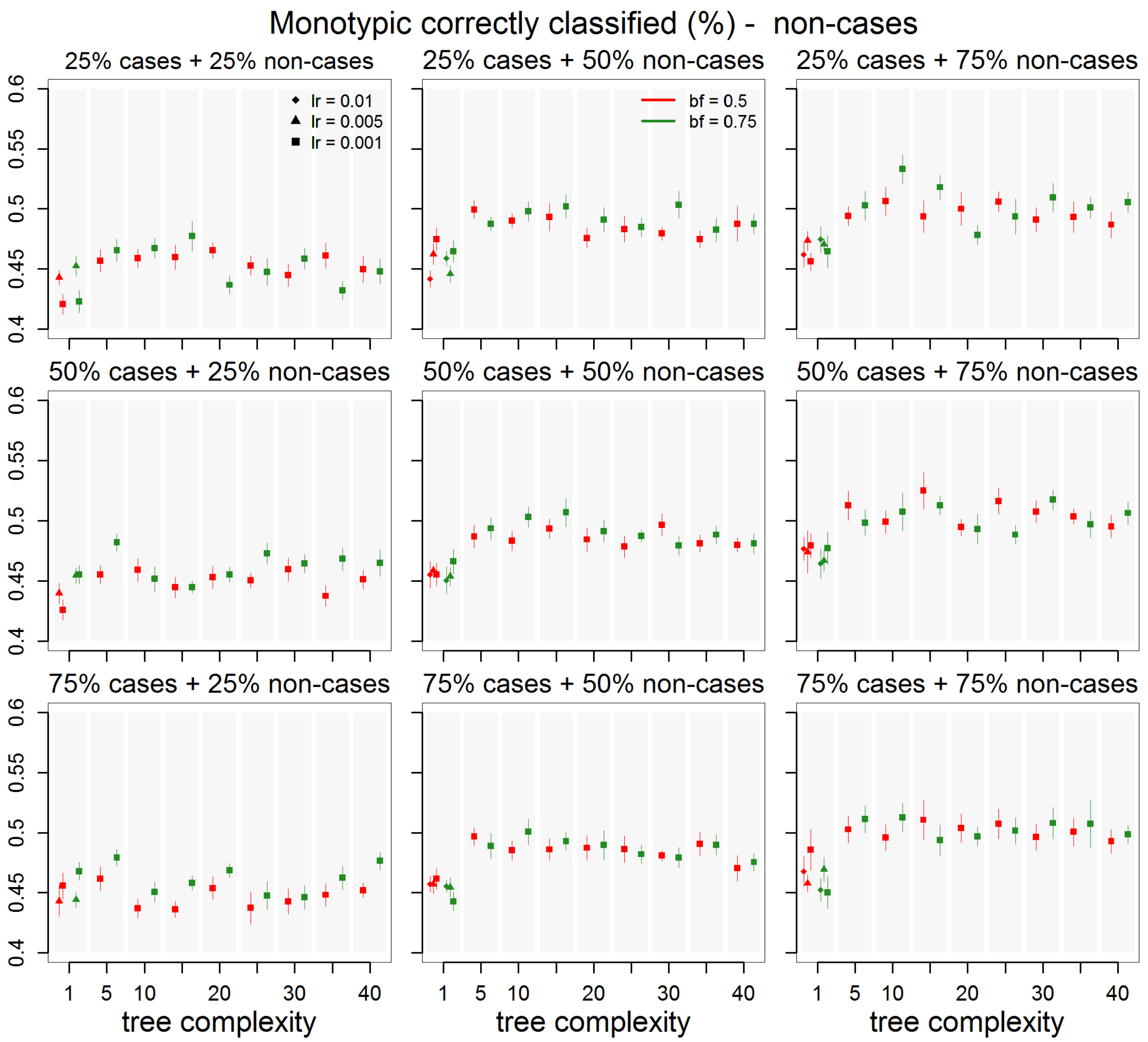

Supplementary Figure 26 Out-of-sample proportion of monotypic non-cases correctly classified (mean and 95% CI) using all combinations of tree complexities (tc = 1, 5, 10, 15, 20, 25, 30, 35, 40), learning rates (lr = 0.01, 0.005, 0.001), bag fractions (bf = 0.5, 0.75) and training sets (see Supplementary Table 1) from 10 randomly sampled training and validation sets (Supplementary section 1.2.2). Only BRT models with at least 1,000 trees are shown.

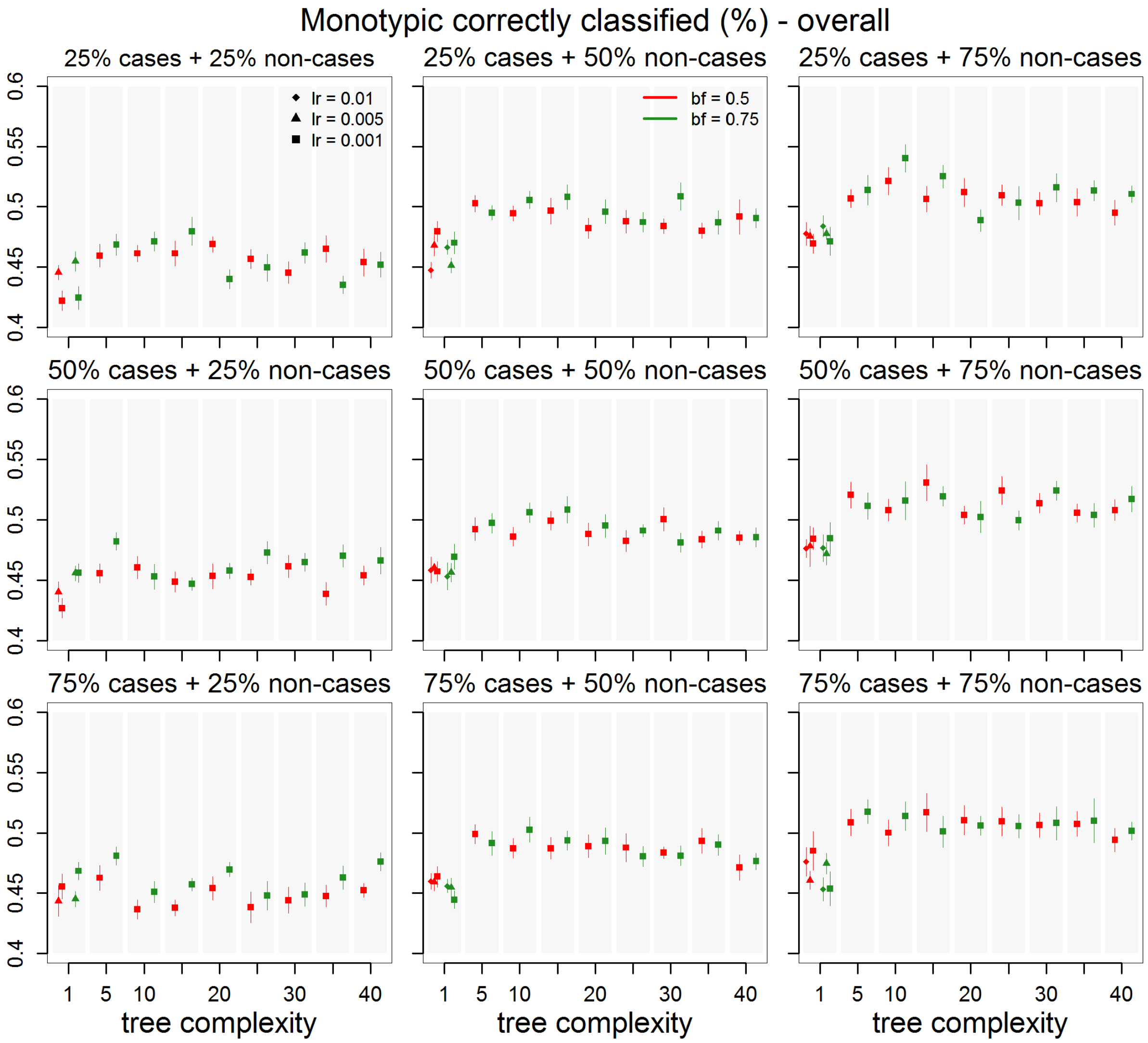

Supplementary Figure 27 Out-of-sample proportion of monotypic cases and non-cases (overall) correctly classified (mean and 95% CI) using all combinations of tree complexities (tc = 1, 5, 10, 15, 20, 25, 30, 35, 40), learning rates (lr = 0.01, 0.005, 0.001), bag fractions (bf = 0.5, 0.75) and training sets (see Supplementary Table 1) from 10 randomly sampled training and validation sets (Supplementary section 1.2.2). Only BRT models with at least 1,000 trees are shown.

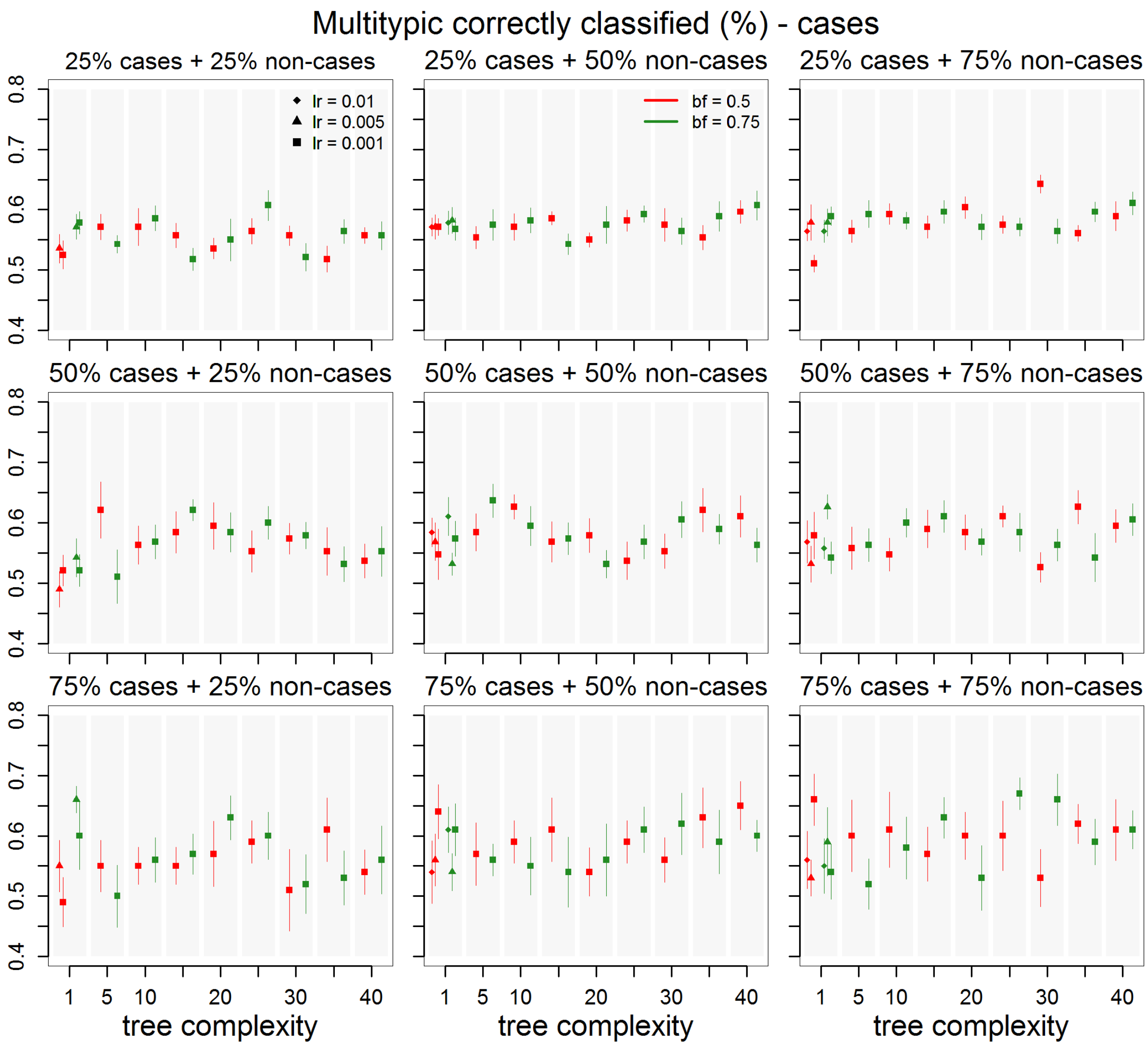

Supplementary Figure 28 Out-of-sample proportion of multitypic cases correctly classified (mean and 95% CI) using all combinations of tree complexities (tc = 1, 5, 10, 15, 20, 25, 30, 35, 40), learning rates (lr = 0.01, 0.005, 0.001), bag fractions (bf = 0.5, 0.75) and training sets (see Supplementary Table 1) from 10 randomly sampled training and validation sets (Supplementary section 1.2.2). Only BRT models with at least 1,000 trees are shown.

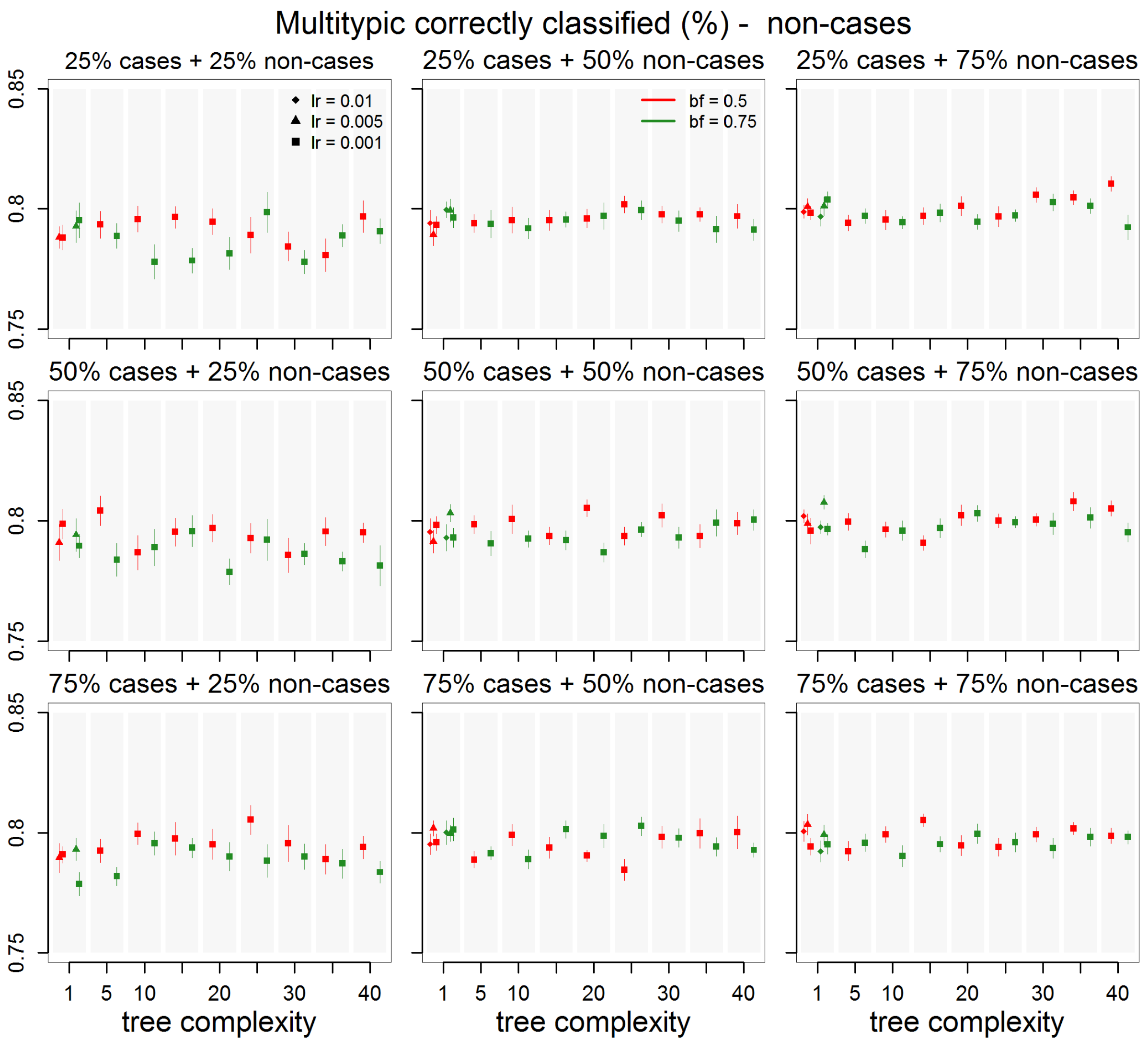

Supplementary Figure 29 Out-of-sample proportion of multitypic non-cases correctly classified (mean and 95% CI) using all combinations of tree complexities (tc = 1, 5, 10, 15, 20, 25, 30, 35, 40), learning rates (lr = 0.01, 0.005, 0.001), bag fractions (bf = 0.5, 0.75) and training sets (see Supplementary Table 1) from 10 randomly sampled training and validation sets (Supplementary section 1.2.2). Only BRT models with at least 1,000 trees are shown.

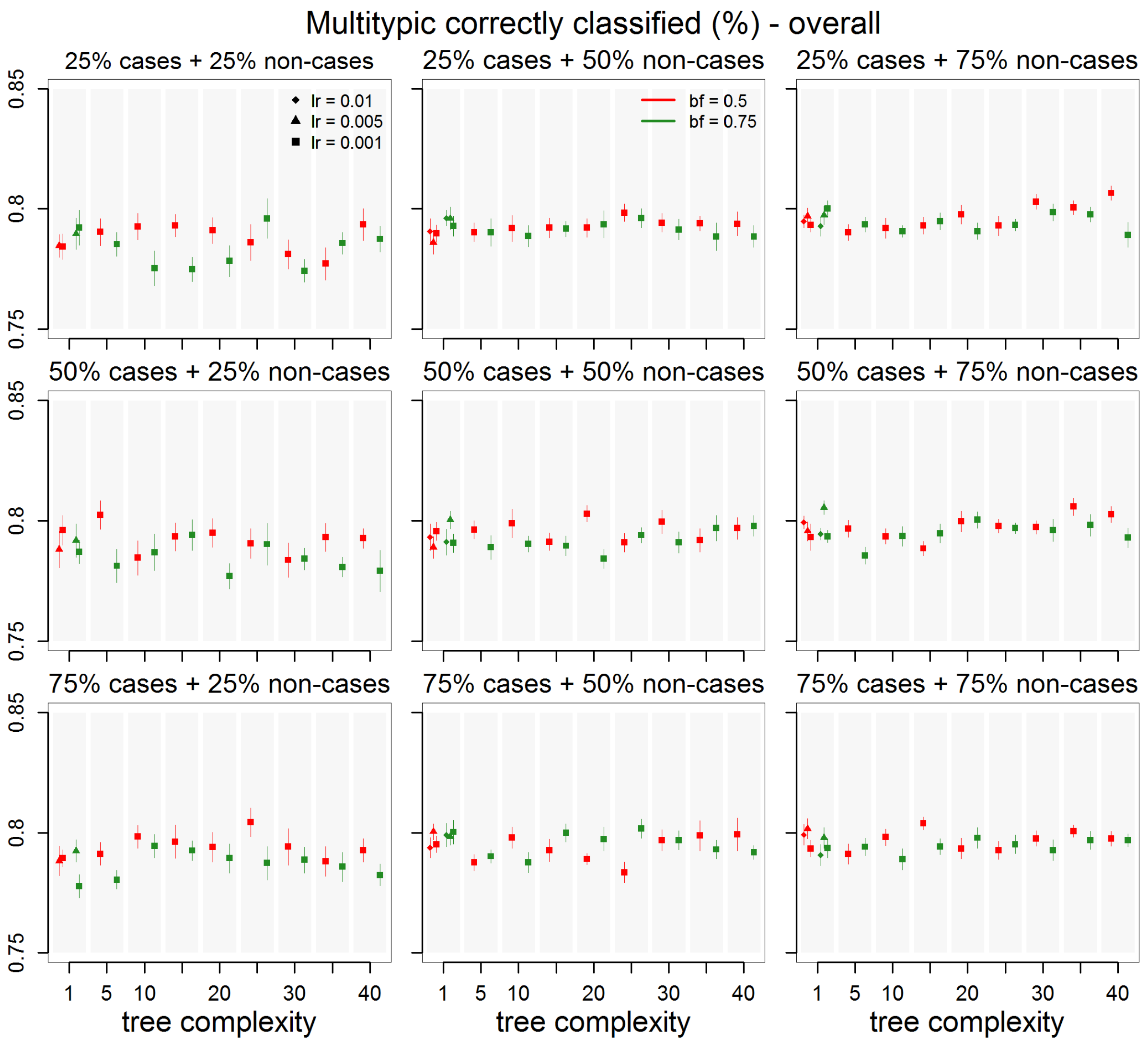

Supplementary Figure 30 Out-of-sample proportion of multitypic cases and non-cases (overall) correctly classified (mean and 95% CI) using all combinations of tree complexities (tc = 1, 5, 10, 15, 20, 25, 30, 35, 40), learning rates (lr = 0.01, 0.005, 0.001), bag fractions (bf = 0.5, 0.75) and training sets (see Supplementary Table 1) from 10 randomly sampled training and validation sets (Supplementary section 1.2.2). Only BRT models with at least 1,000 trees are shown.

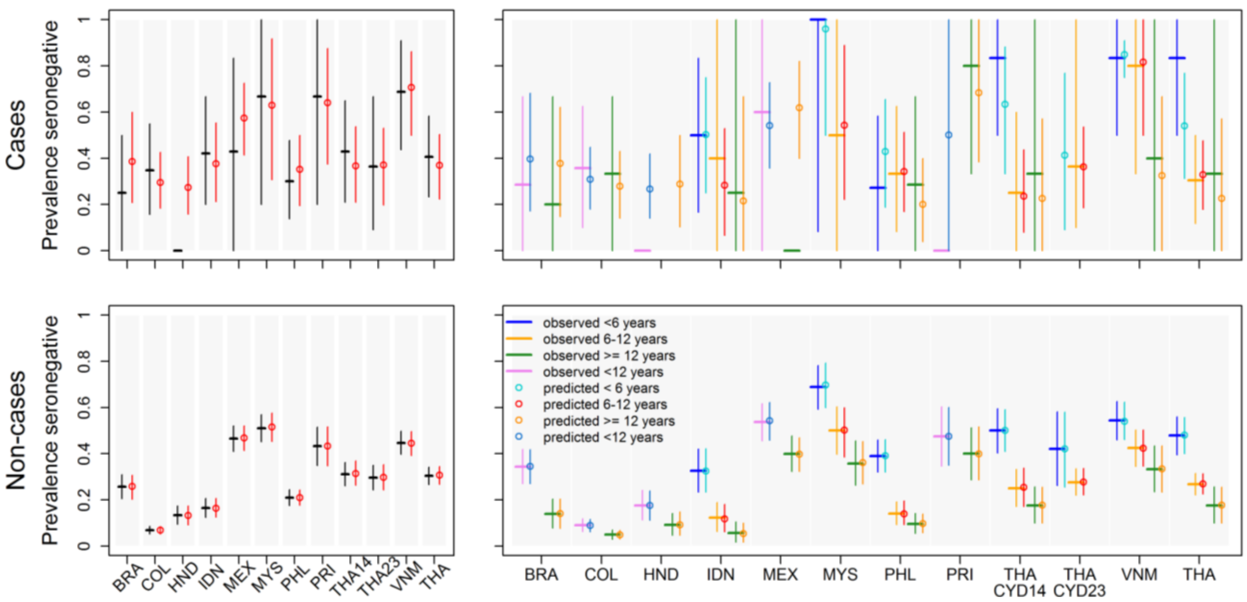

Supplementary Figure 31 Mean and 95% CI of the observed and estimated proportion of seronegative subjects among cases (first row) and non-cases (second row) by country (first column) and by country and age-group (second column) obtained from 1,000 realisations of the BRT model using 5-fold cross-validation, tc = 15, lr = 0.001, bf = 0.75 and by randomly sampling 75% of non-cases and 75% of cases to build the training set.

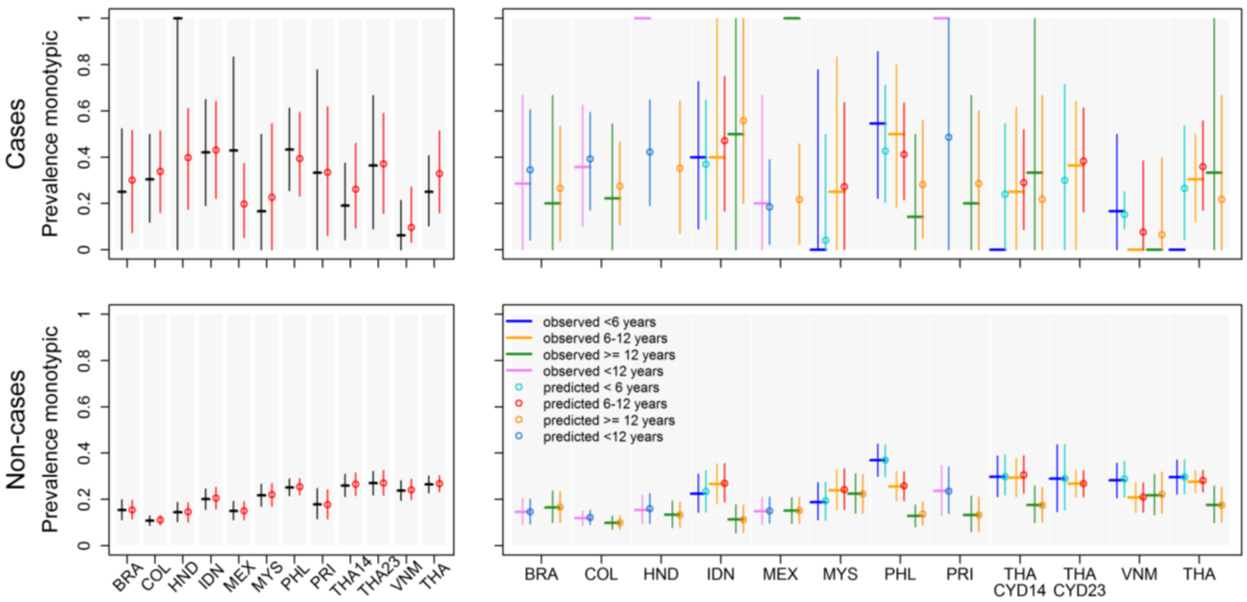

Supplementary Figure 32 Mean and 95% CI of the observed and estimated proportion of monotypic subjects among cases (first row) and non-cases (second row) by country (first column) and by country and age-group (second column) obtained from 1,000 realisations of the BRT model using 5-fold cross-validation, tc = 15, lr = 0.001, bf = 0.75 and by randomly sampling 75% of non-cases and 75% of cases to build the training set.

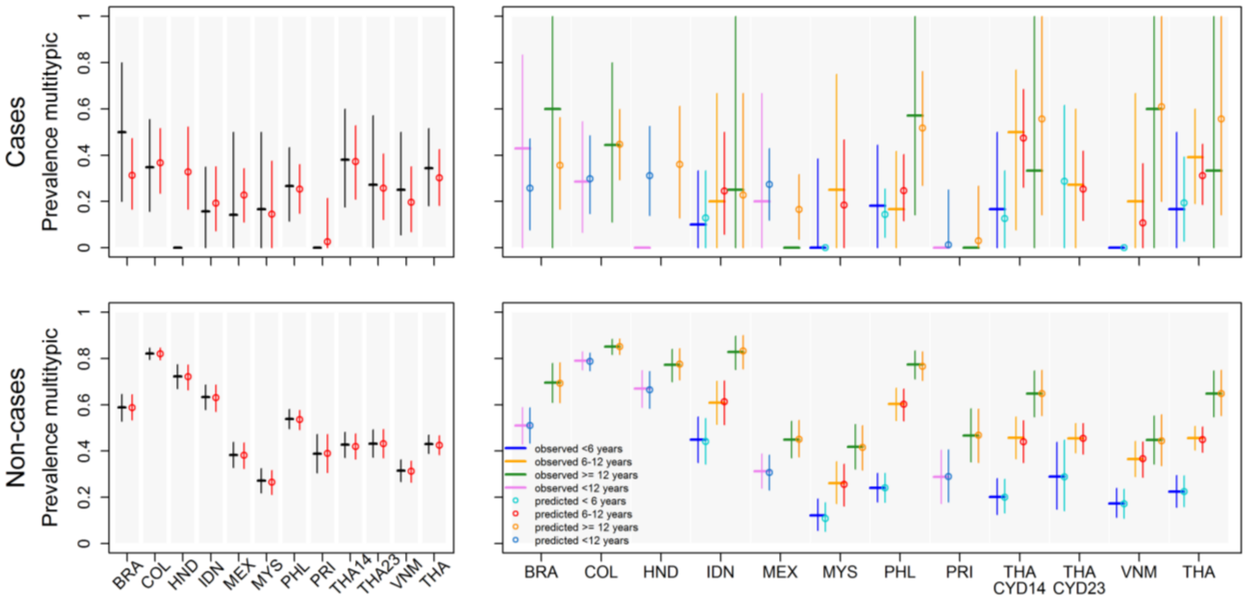

Supplementary Figure 33 Mean and 95% CI of the observed and estimated proportion of multitypic subjects among cases (first row) and non-cases (second row) by country (first column) and by country and age-group (second column) obtained from 1,000 realisations of the BRT model using 5-fold cross-validation, tc = 15, lr = 0.001, bf = 0.75 and by randomly sampling 75% of non-cases and 75% of cases to build the training set.

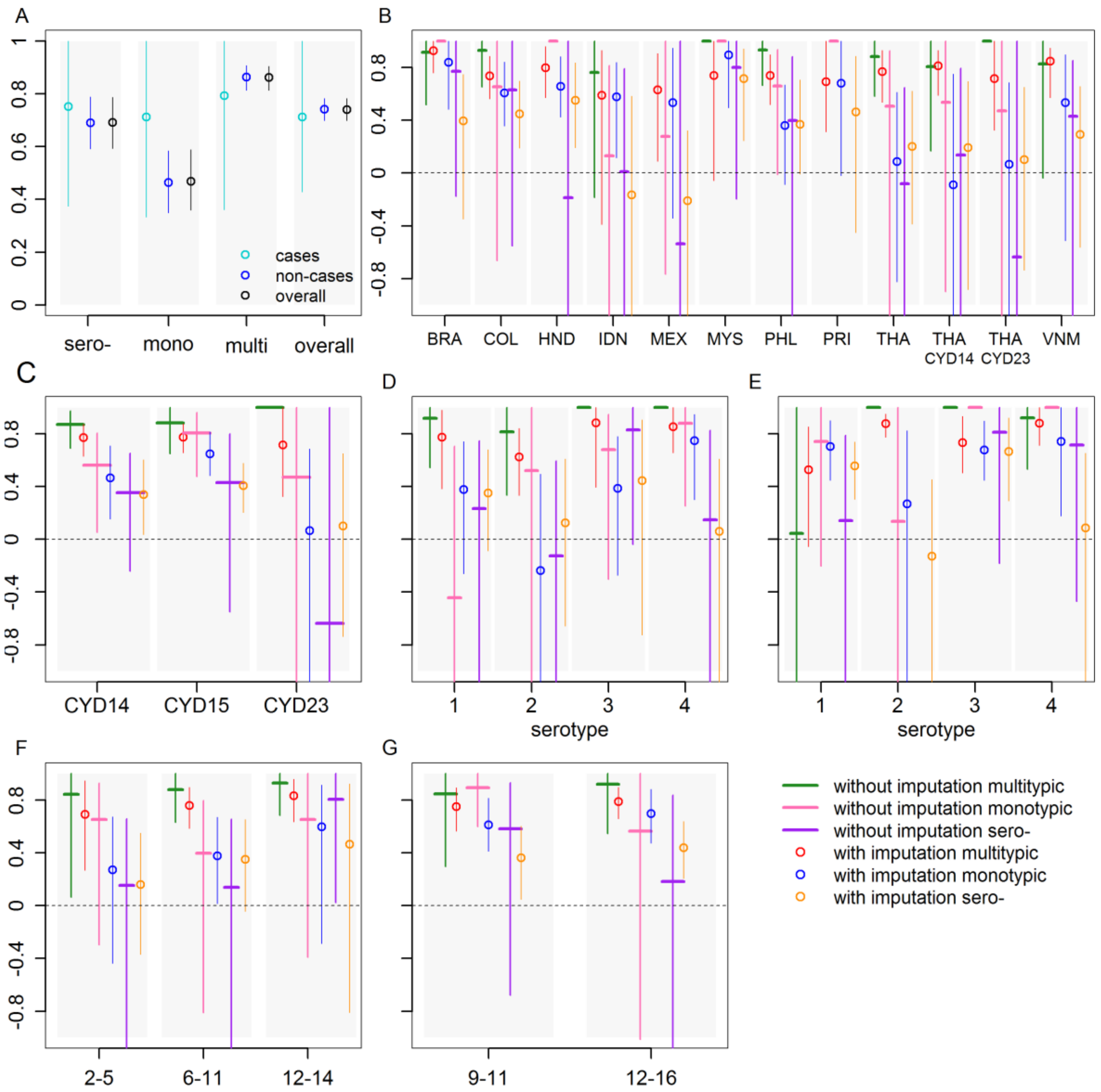

Supplementary Figure 34 Mean and 95% CI of the vaccine efficacy estimates generated with and without imputed data for the subjects with baseline seronegative, monotypic and multitypic PRNT50 titre profiles separately. Estimates were obtained from 1,000 realisations of the final BRT model achieving at least 30% accuracy for cases and non-cases, trained on 75% of non-cases and 75% of cases and using 5-fold cross-validation, tc = 15, lr = 0.001, bf = 0.75. A) proportion of imputations correct classifications among seronegative (sero-), monotypic (mono), multitypic (multi) and overall (i.e. seronegative, monotypic and multitypic) cases, non-cases and overall (cases and non-cases); B) vaccine efficacy estimates by country for baseline seronegative, monotypic and multitypic subjects (BRA = Brazil, OL = Colombia, HND = Honduras, IDN = Indonesia, MEX = Mexico, MYS = Malaysia, PHL = Philippines, PRI = Puerto Rico, THA = Thailand, VNM = Vietnam). Efficacy estimates for Thailand were generated using the phse-2b (CYD23) and the phase-3 (CYD14) data separately and combined; C) vaccine efficacy estimates by trial (CYD14 = phase-3 trial in South-east Asia, CYD15 = phase-3 trial in Latin America, CYD23 = phase-2b trial in Thailand) for baseline seronegative, monotypic and multitypic subjects; D) vaccine efficacy estimates by serotype in South-east Asia (i.e. using CYD14 and CYD23 combined) for baseline seronegative, monotypic and multitypic subjects; E) vaccine efficacy estimates by serotype in Latin America (i.e. using CYD15) for baseline seronegative, monotypic and multitypic subjects; F) vaccine efficacy estimates by age in South-east Asia (i.e. using CYD14 and CYD23) for baseline seronegative, monotypic and multitypic subjects; G) vaccine efficacy estimates by age in Latin America (i.e. using CYD15) for baseline seronegative, monotypic and multitypic subjects.

2.8.5 Vaccine efficacy estimates using alternative definitions of monotypic and multitypic PRNT50 profiles

We tested the effect of using alternative definitions for monotypic and multitypic PRNT50 profiles. The results in this section were obtained by defining subjects with only one PRNT50 titre > 10 as monotypic, and subjects with more than one PRNT50 titre > 10 as multitypic.

Using these definitions, we found that the optimal hyper-parameterisation was obtained for *tc* = 20, *lr* = 0.001, *bf* = 0.5 by sampling 75% of the cases and 75% of the non-cases. Using this hyper-parameterisation, we estimated the prevalence of seronegative, monotypic and multitypic profiles shown in Supplementary Figures 35-37 and the efficacy estimates shown in Supplementary Figures 38-39.

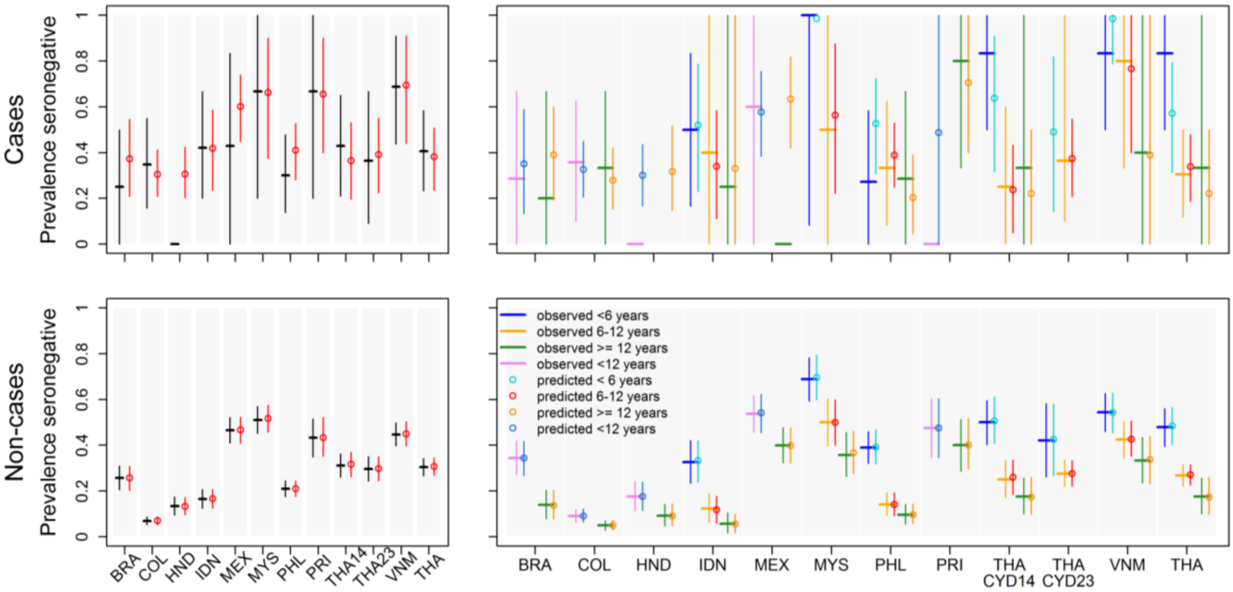

Supplementary Figure 35 Mean and 95% CI of the observed and estimated proportion of seronegative subjects among cases (first row) and non-cases (second row) by country (first column) and by country and age-group (second column) obtained from 1,000 realisations of the BRT model using 5-fold cross-validation, tc = 20, lr = 0.001, bf = 0.5 and by randomly sampling 75% of non-cases and 75% of cases to build the training set.

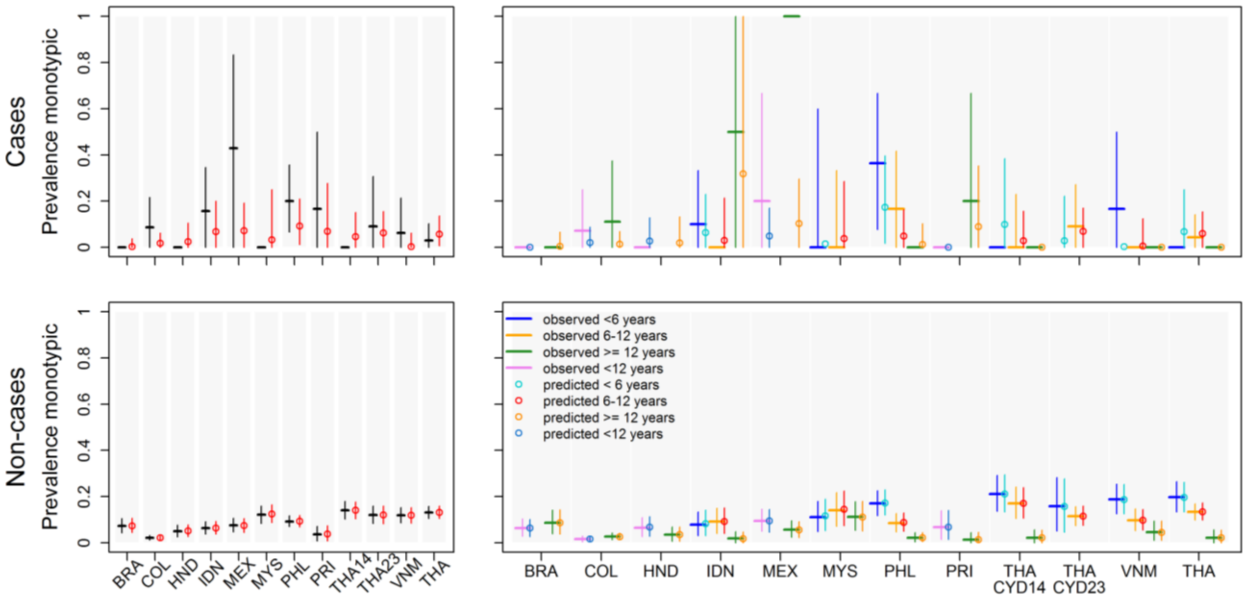

Supplementary Figure 36 Mean and 95% CI of the observed and estimated proportion of monotypic subjects among cases (first row) and non-cases (second row) by country (first column) and by country and age-group (second column) obtained from 1,000 realisations of the BRT model using 5-fold cross-validation, tc = 20, lr = 0.001, bf = 0.5 and by randomly sampling 75% of non-cases and 75% of cases to build the training set.

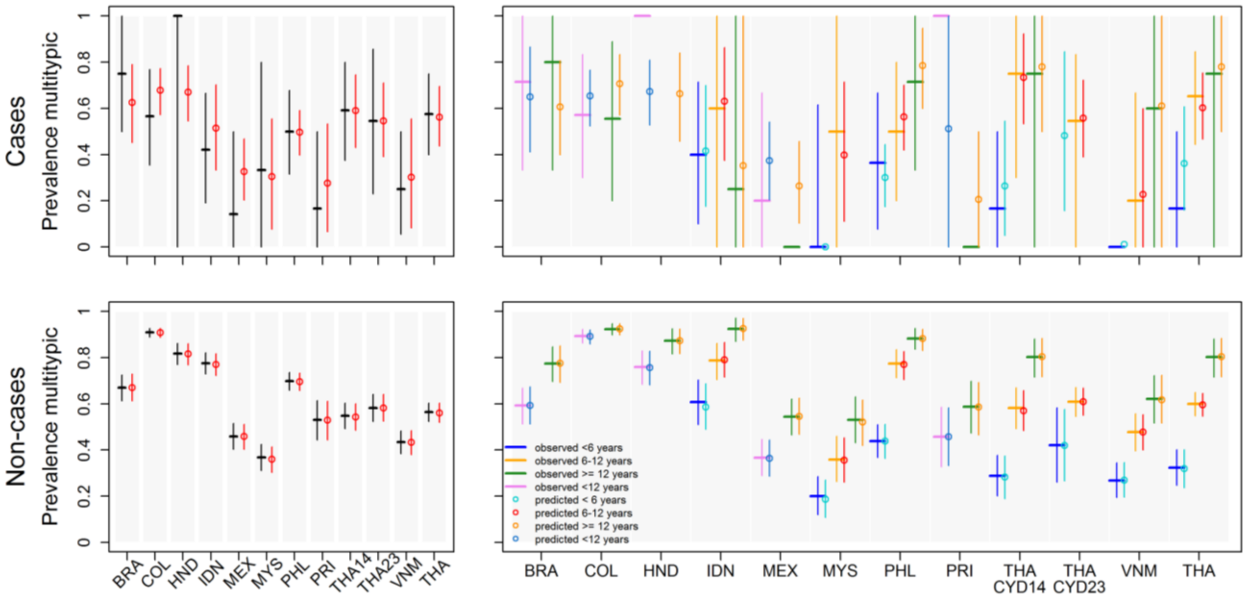

Supplementary Figure 37 Mean and 95% CI of the observed and estimated proportion of multitypic subjects among cases (first row) and non-cases (second row) by country (first column) and by country and age-group (second column) obtained from 1,000 realisations of the BRT model using 5-fold cross-validation, tc = 20, lr = 0.001, bf = 0.5 and by randomly sampling 75% of non-cases and 75% of cases to build the training set.

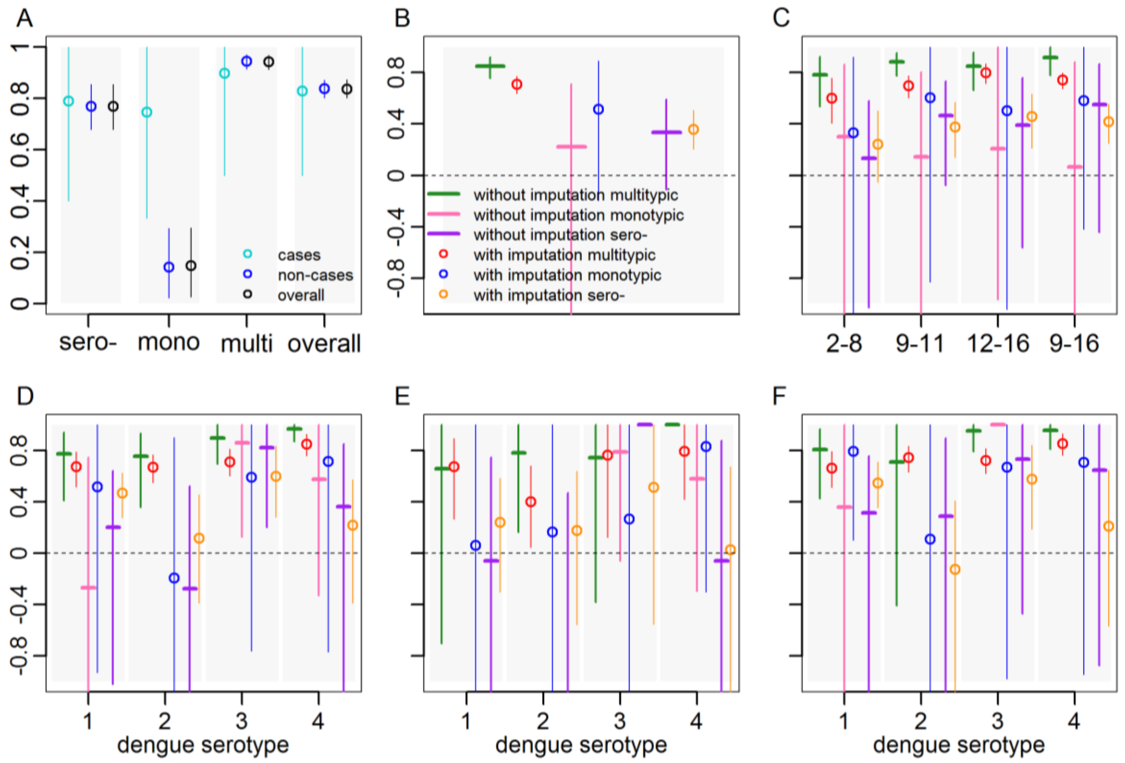

Supplementary Figure 38 Mean and 95% CI of the vaccine efficacy estimates generated with and without imputed data for the subjects with baseline seronegative, monotypic and multitypic PRNT50 titre profiles separately. Estimates were obtained from 1,000 realisations of the final BRT model achieving at least 30% accuracy for the cases, trained on 75% of non-cases and 75% of cases and using 5-fold cross-validation, tc = 20, lr = 0.001, bf = 0.5. A) proportion of imputations correct classifications among seronegative (sero-), monotypic (mono), multitypic (multi) and overall (i.e. seronegative, monotypic and multitypic) cases, non-cases and overall (cases and non-cases); B) vaccine efficacy estimates for baseline multitypic, monotypic and seronegative subjects separately; C) vaccine efficacy estimates for baseline multitypic, monotypic and seronegative subjects by age, using 2-8, 9-11, 12-16 and 9-16 years age-groups; D) vaccine efficacy estimates by serotype for baseline multitypic, monotypic and seronegative subjects over all ages (2-16 years); E) vaccine efficacy estimates by serotype for baseline multitypic, monotypic and seronegative subjects 2-8 years old; F) vaccine efficacy by serotype for baseline multitypic, monotypic and seronegative subjects 9-16 years old.

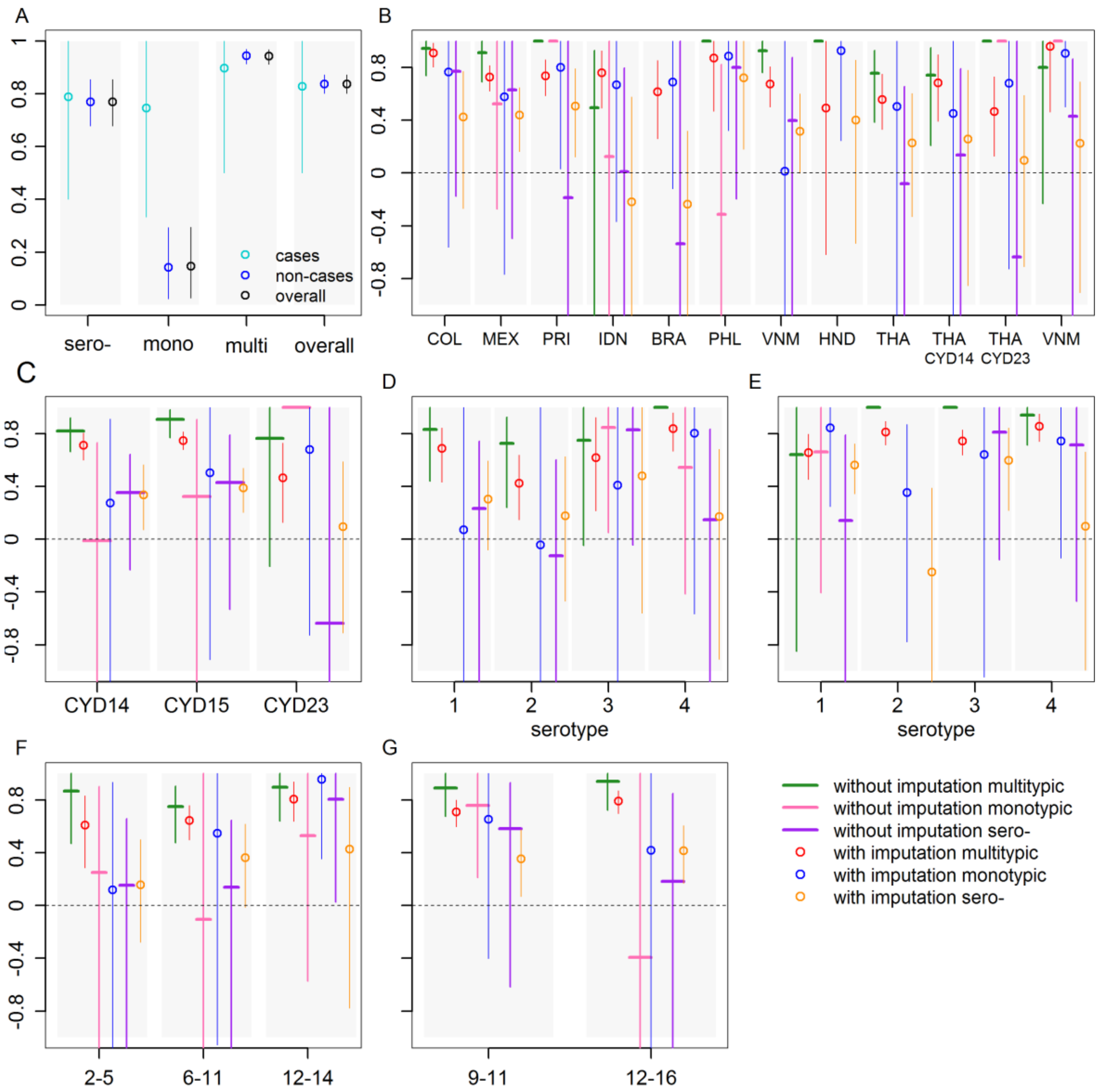

Supplementary Figure 39 Mean and 95% CI of the vaccine efficacy estimates generated with and without imputed data for the subjects with baseline seronegative, monotypic and multitypic PRNT50 titre profiles separately. Estimates were obtained from 1,000 realisations of the final BRT model achieving at least 30% accuracy for the cases, trained on 75% of non-cases and 75% of cases and using 5-fold cross-validation, tc = 20, lr = 0.001, bf = 0.5. A) proportion of imputations correct classifications among seronegative (sero-), monotypic (mono), multitypic (multi) and overall (i.e. seronegative, monotypic and multitypic) cases, non-cases and overall (cases and non-cases); B) vaccine efficacy estimates by country for baseline seronegative, monotypic and multitypic subjects (BRA = Brazil, OL = Colombia, HND = Honduras, IDN = Indonesia, MEX = Mexico, MYS = Malaysia, PHL = Philippines, PRI = Puerto Rico, THA = Thailand, VNM = Vietnam). Efficacy estimates for Thailand were generated using the phse-2b (CYD23) and the phase-3 (CYD14) data separately and combined; C) vaccine efficacy estimates by trial (CYD14 = phase-3 trial in South-east Asia, CYD15 = phase-3 trial in Latin America, CYD23 = phase-2b trial in Thailand) for baseline seronegative, monotypic and multitypic subjects; D) vaccine efficacy estimates by serotype in South-east Asia (i.e. using CYD14 and CYD23 combined) for baseline seronegative, monotypic and multitypic subjects; E) vaccine efficacy estimates by serotype in Latin America (i.e. using CYD15) for baseline seronegative, monotypic and multitypic subjects; F) vaccine efficacy estimates by age in South-east Asia (i.e. using CYD14 and CYD23) for baseline seronegative, monotypic and multitypic subjects; G) vaccine efficacy estimates by age in Latin America (i.e. using CYD15) for baseline seronegative, monotypic and multitypic subjects.

2.8.6 Visualisation of post-dose 3 PRNT50 titres for seronegative, monotypic and multitypic profiles

Supplementary Figures 40 and 41 show the observed PD3 PRNT50 titres of the subjects with baseline seronegative, monotypic and multitypic profile, using the classifications defined in the main text (see Methods) and in Supplementary section 2.8.5, respectively.

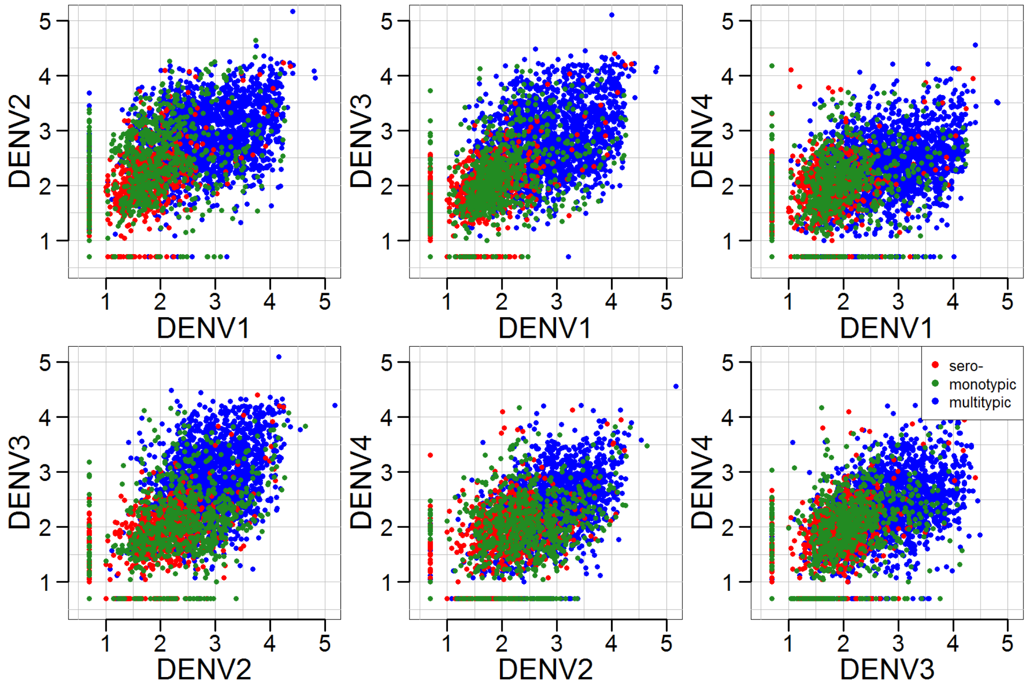

Supplementary Figure 40 PD3 PRNT50 titres of the subjects in CYD14, CYD15 and CYD23, using the classification defined in the main text. Seronegative (sero-) = subjects with all four baseline PRNT50 titres < 10; monotypic = subjects with one baseline PRNT50 titres > 10 or, if more than one > 10, only one > 80; multitypic = subjects with more than one PRNT50 titre > 10 and more than one > 80).

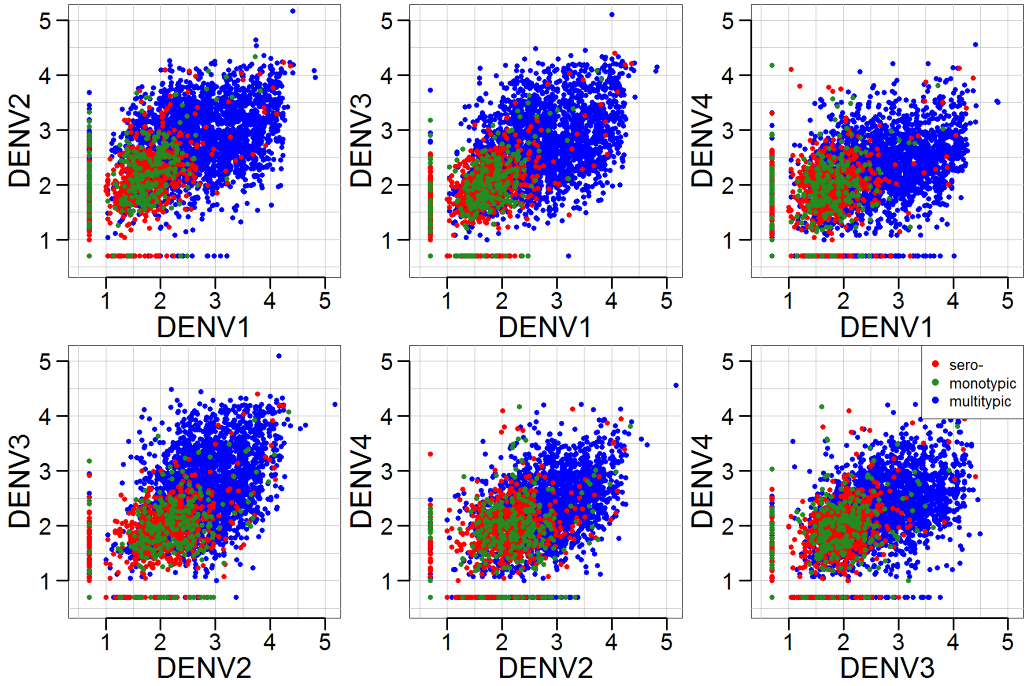

Supplementary Figure 41 PD3 PRNT50 titres of the subjects in CYD14, CYD15 and CYD23, using the classification defined in Supplementary section 2.8.5. Seronegative (sero-) = subjects with all four baseline PRNT50 titres < 10; monotypic = subjects with one baseline PRNT50 titres > 10; multitypic = subjects with more than one PRNT50 titre > 10.

2.9. Robustness of imputation using alternative machine learning methods to BRT

In our main analysis we chose BRT for data imputation. This choice was dictated by its increasing application and success in similar statistical epidemiology problems[9-14] its ease of application and accessibility[15], its excellent performance observed in several comparative analyses applied to diverse datasets, including a comprehensive comparison conducted on a complex classification problem[16] and its ability to incorporate incomplete or missing data (as opposed to other algorithms that require complete data both for training and prediction). This latter was an important consideration for our dataset, given that 18% (28 out 155) of the cases and 2.5% (106 out of 4096) of the non-cases with observed baseline serostatus had incomplete data on at least one of the other predictors. We preferred not to utilise automatic imputation techniques to fill in for missing data, e.g. with the average observed across the dataset or other algorithms implemented in other machine learning methods.

In this section we report the results of a comparison between BRT's performance with other well-established alternatives such as generalised linear models (GLM), random forest (RF), support vector machine (SVM) and neural network (NN). We did not utilise automatic imputation technique to fill in for missing data in the predictors for the machine learning algorithms unable to handle incomplete data and in these cases we restricted the model training and evaluation to the subjects with complete data on the predictors. In this analysis we optimised the tuning parameters of each method using random search. Specifically, for each method we sampled 1,000 parameterisations on the numbers of cases and non-cases with complete information on the baseline serostatus used for training (integer distribution between 39 and 116 for cases and between 1,024 and 3,072 for non-cases) and in addition:

- for RF, we also randomly sampled the number of trees to grow (integer distribution between 500 and 5,000) and the number of predictors randomly sampled as candidates at each split (integer distribution between 3 and 11);
- for SVM, we also randomly sampled the kernel ('linear', 'sigmoid', 'polynomial' or 'radial'), the degree of the kernel for polynomial kernels (integer distribution between 1 and 10), the gamma kernel parameter (uniformly sampled between 0.01 and 100 on the logarithmic scale), the coef0 parameter used for polynomial and sigmoidal kernels (uniformly sampled between 1 and 1,000 on the logarithmic scale), the cost of constraints violations (uniformly sampled between 0.01 and 10,000 on the logarithmic scale) and class weights (uniformly sampled between 0.5 and 1 for baseline seronegative subjects, implying a higher weight than for baseline seropositive subjects);
- for NN (single hidden layer neural network), we also randomly sampled the units in the hidden layer (uniformly distributed between 1 and 20) and the regularisation parameter to avoid over-fitting (uniformly distributed between 0.00001 and 0.5).

GLM and NN were implemented using the 'caret' package. RF was implemented using the 'randomForest' package and SVM (C-classification type) was implemented using the 'e1071' package. For consistency with BRT, we used 10-fold cross-validation for all methods and we optimised two distinct cut-off probability thresholds for cases and non-cases separately for all algorithms except support vector machine, which by construction is built on the concept of distance and lacks a probabilistic implementation (although probabilistic interpretations have been proposed[17,18]). We used two distinct cut-off probability thresholds for cases and non-cases because this allowed to achieve a balanced sensitivity and specificity among cases, as opposed to using a single cut-off threshold, which resulted in an average 14% lower sensitivity than specificity for the cases (see Supplementary section 1.2.2).

Supplementary Figure 42 shows the sensitivity, specificity and proportion of predictions correctly classified among cases, non-cases and overall using each of these methods and the optimal

parameterisation found using random search. We find that while BRT, GLM, NN and RF performed similarly in terms of sensitivity, specificity and percentage of predictions correctly classified overall and among non-cases, RF showed a slightly higher predictive performance among cases (77%) as compared to BRT (74.5%), NN (72%), and GLM (68%). SVM produced higher sensitivities overall (90%) and among non-cases (90%) and higher specificity (86%) among cases than BRT, GLM, NN and RF but lower specificity (83%) overall and among non-cases and lower sensitivity (65%) among cases than BRT, GLM, NN and RF.

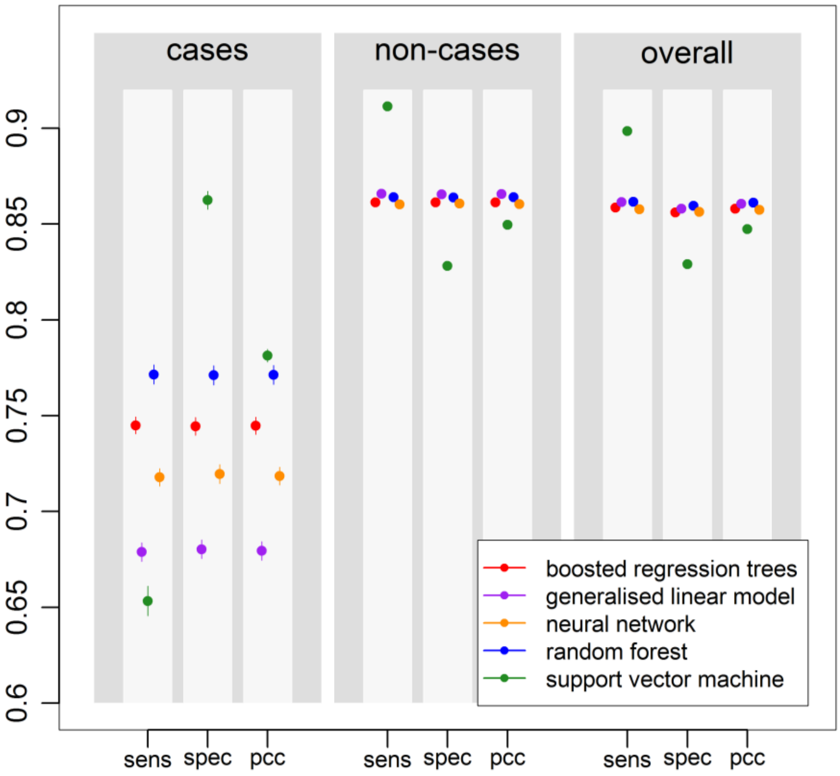

Supplementary Figure 42 Out-of-sample sensitivity (proportion of baseline seropositive subjects correctly classified), specificity (proportion of baseline seronegative subjects correctly classified) and proportion of correct classifications among cases, non-cases and overall using BRT, GLM, NN, RF and SVM models. Each model was parametrised using the respective optimal hyper-parameterisation found using random hyper-parameter search and the optimal size of the training sets. Each method was built on 100 randomly drawn training and validation sets, using the algorithm described in Supplementary section 1.2.2.

We then assessed the impact of the small increase in predictive performance observed among cases using RF compared with BRT on the vaccine efficacy estimates. For consistency with the estimates obtained using BRT, we first conducted automatic feature selection on the RF model (using the optimal parameterisation) and then used the final RF model (i.e. with only the important variables included) to generate the vaccine efficacy estimates with bootstrapping.

We used the 'VSURF' package for automatic feature selection using the optimal parameterisation and 100 realisations of the RF model and found that trial, gender, serotype and case occurrence were retained only in 39%, 36%, 1% and 0% of the realisations respectively in the preliminary elimination and ranking step (all other predictors were retained in > 90% of the realisations). The same variables were discarded also in the interpretation and prediction steps of the VSURF procedure. Supplementary Table 13 shows the average variable importance of the predictors included in the full RF model (i.e. with all available predictors included) and in the final model (i.e. with only the predictive variables included, having discarded case occurrence, serotype, gender and trial).

Predictors	Full RF model	Final RF model
PD3 PRNT50 titre against DENV1	12.18	12.86
PD3 PRNT50 titre against DENV2	11.98	12.26
PD3 PRNT50 titre against DENV3	9.76	9.94
PD3 PRNT50 titre against DENV4	6.60	6.80
Country	4.64	4.97
Age	3.94	4.40
Arm	3.81	3.72
Trial	0.63	-
Gender	0.53	-
Serotype	0.25	-
Case	0.07	-

Supplementary Table 14 Variable importance of the predictors used in the full and final RF models using 10-fold cross-validation and the optimal hyper-parameterisation, where each model was built on 100 randomly drawn training and validation sets, using the algorithm described in Supplementary section 1.2.2. The full model used 11 predictors and the final model used 7 predictors. The time interval between dose 3 to symptoms onset was not used in RF because this was missing for all non-cases in the dataset.

Finally, we used the final RF model to estimate the vaccine efficacy. Supplementary Figure 43 shows a comparison of the results obtained using the final BRT and final RF models both in terms of accuracy and vaccine efficacy by baseline serostatus, age and serotype.

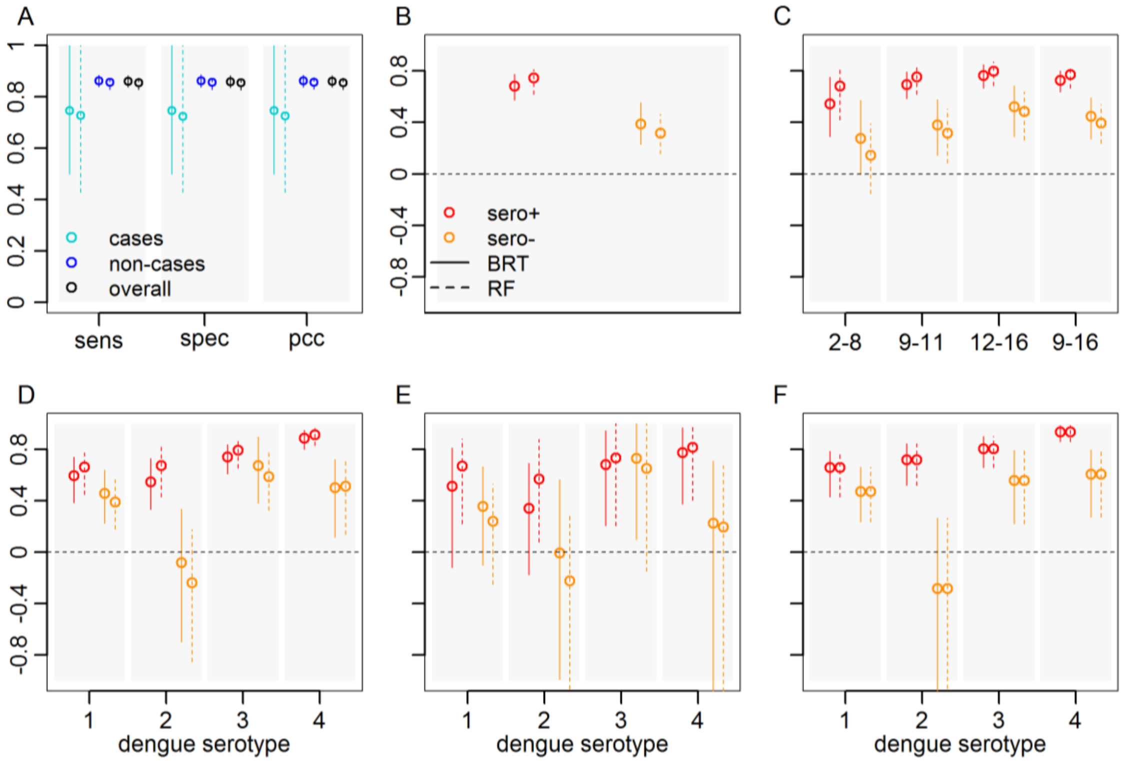

Supplementary Figure 43 Mean and 95% CI of the vaccine efficacy estimates generated with imputed data for baseline seronegative (sero-) and baseline seropositive (sero+) subjects separately using BRT and RF. Estimates were obtained from 1,000 realisations of the final BRT and RF models. A) sensitivity (sens), specificity (spec) and proportion of correct classifications (pcc) among cases, non-cases and overall; B) vaccine efficacy estimates for baseline seropositive and baseline seronegative subjects separately; C) vaccine efficacy estimates for baseline seropositive and baseline seronegative subjects by age using 2-8, 9-11, 12-16 and 9-16 years age-groups ; D) vaccine efficacy estimates by serotype for baseline seropositive and baseline seronegative subjects of all ages (2-16 years); E) vaccine efficacy estimates by serotype for baseline

seropositive and baseline seronegative subjects 2-8 years old; F) vaccine efficacy estimates by serotype for baseline seropositive and baseline seronegative subjects 9-16 years old.

Supplementary Figure 43A shows that the accuracy measures obtained with BRT and RF are consistent (both among cases, non-cases and overall). Supplementary Figures 43B-F show that the vaccine efficacy estimates by baseline serostatus, age and serotype are robust to the use of BRT or RF as imputation method.

With the final RF model and using 2-8, 9-11 and 12-16 years age-groups we found a significant age-trend among baseline seronegative subjects giving 3.8% [95% CI: 0.4, 7.2%] (p-value = 0.04) increase in VE for each year-increase in age. The age-trends in vaccine efficacy estimated among baseline seronegative subjects using RF are consistent with the estimates obtained with BRT. These results provide further evidence of the robustness of the estimates presented in the main analysis.

3. Supplementary References

1. Sabchaeron, A. et al. Protective efficacy of the recombinant, live-attenuated, CYD tetravalent dengue vaccine in Thai schoolchildren: a randomised, controlled phase 2b trial. Lancet; **380**(9853): 1559-67 (2012).
2. Capeding, M.R. et al. Clinical efficacy and safety of a novel tetravalent dengue vaccine in healthy children in Asia: A phase 3, randomised, observer-masked, placebo-controlled trial. Lancet; **384**(9951): 1358–1365 (2014).
3. Villar, L. et al. Efficacy of a Tetravalent Dengue Vaccine in Children in Latin America. N. Engl. J. Med.; **372**(2):113-23 (2014).
4. Elith J, Leathwick JR, Hastie T, A working guide to bossted regression trees. J Anim Ecol.; **77**(4): 802-13 (2008).
5. Friedman JH. Stochastic gradient boosting. Computational Statistics and Data Analysis; **38**: 367–378 (2002).
6. Robert J. Hijmans, Steven Phillips, John Leathwick and Jane Elith. dismo: Species Distribution Modeling. R package version 1.1-1. https://CRAN.R-project.org/package=dismo (2016).
7. Freeman EA and Moisen G. PresenceAbsence: An R Package for Presence-Absence Model Analysis. Journal of Statistical Software, **23**(11):1-31. http://www.jstatsoft.org/v23/i11 (2008).
8. Greg Ridgeway with contributions from others. gbm: Generalized Boosted Regression Models. R package version 2.1.1. https://CRAN.R-project.org/package=gbm (2015).
9. Bhatt, S. et al. The global distribution and burden of dengue. Nature; **496**: 504–7 (2013).
10. Gilbert, B. et al. Predicting the risk of avian influenza A H7N9 infection in live-poultry markets across Asia. Nat Commun.; **5**: 4116 (2014).
11. Kraemer, M.U.G. et al. The global distribution of the arbovirus vectors Aedes aegypti and Ae. albopictus. eLife; **4**: e08347 (2015).
12. Sinka, M.E. et al. A global map of dominant malaria vectors. Parasit Vectors; **5**:69 (2012).
13. Sinka, M.E. et al. The dominant Anopheles vectors of human malaria in the Asia-Pacific region: occurrence data, distribution maps and bionomic précis. Parasit Vectors; **4**:89 (2011).
14. Pigott, D.M. et al. Mapping the zoonotic niche of Ebola virus disease in Africa. eLife. **3**: e04395 (2014).
15. Elith J, Leathwick JR, Hastie T, A working guide to boosted regression trees. J Anim Ecol.; **77**(4): 802-13 (2008).
16. Elith, J. et al. Novel methods improve prediction of species'distributions from occurrence data. Ecography; **29**: 129-151 (2006).
17. Sollich P. Probabilistic methods for Support Vector Machines. Adv Neural Inf Process Syst; **1**:349-355 (2000).
18. Tao Q, Wu G, Wang F, Wang J. Posterior probability support vector machines for unbalanced data. IEEE Trans. Neural Netw.; **16**(6): 1561-1573 (2005).
